# 3D sub-nanometer analysis of glucose in an aqueous solution by cryo-atom probe tomography

**DOI:** 10.1038/s41598-021-90862-8

**Published:** 2021-06-02

**Authors:** T. M. Schwarz, C. A. Dietrich, J. Ott, E. M. Weikum, R. Lawitzki, H. Solodenko, E. Hadjixenophontos, B. Gault, J. Kästner, G. Schmitz, P. Stender

**Affiliations:** 1 Chair of Materials Physics, Institute for Materials Science, University of Stuttgart, Heisenbergstr. 3, 70569 Stuttgart, Germany; 2 Institute for Theoretical Chemistry, University of Stuttgart, Pfaffenwaldring 55, 70569 Stuttgart, Germany; 3 Max-Planck-Institut Für Eisenforschung, Max-Planck-Str. 1, 40237 Düsseldorf, Germany; 4 Department of Materials, Royal School of Mines, Imperial College, Prince Consort Road, London, SW7 2BP UK

**Keywords:** Techniques and instrumentation, Characterization and analytical techniques, Microscopy, Materials science, Fluids

## Abstract

Atom Probe Tomography (APT) is currently a well-established technique to analyse the composition of solid materials including metals, semiconductors and ceramics with up to near-atomic resolution. Using an aqueous glucose solution, we now extended the technique to frozen solutions. While the mass signals of the common glucose fragments C_*x*_H_*y*_ and C_*x*_O_*y*_H_*z*_ overlap with (H_2_O)_*n*_H from water, we achieved stoichiometrically correct values via signal deconvolution. Density functional theory (DFT) calculations were performed to investigate the stability of the detected pyranose fragments. This paper demonstrates APT’s capabilities to achieve sub-nanometre resolution in tracing whole glucose molecules in a frozen solution by using cryogenic workflows. We use a solution of defined concentration to investigate the chemical resolution capabilities as a step toward the measurement of biological molecules. Due to the evaporation of nearly intact glucose molecules, their position within the measured 3D volume of the solution can be determined with sub-nanometre resolution. Our analyses take analytical techniques to a new level, since chemical characterization methods for cryogenically-frozen solutions or biological materials are limited.

## Introduction

Atom probe tomography (APT)^1–5^ has become a key technique to investigate chemical structures at the near-atomic scale across a broad variety of materials systems^6^. Depending on the controlled removal of individual atoms from a sharp needle-like emitter in the form of ions, this destructive method transforms the studied sample volume into a 3D computer model with individual ions characterized chemically. One of the current trends in APT is to gain a deeper understanding of soft matter in general and liquids in particular^7–16^. While thin layers of ice, created by condensed vapour on metallic tips, have been studied previously^17–21^, the newly available cryo-transfer systems^22,23^ facilitate the preparation and transport of APT specimens from frozen liquids with large sampled volumes. Such a step-change in capability may enable fundamental atomic-scale studies of solutions, bio(macro)molecules in their natural environment, and potentially catalytic reactions as well as other liquid–solid interfaces, e.g., wet corrosion.

Challenges arising from analysing soft matter by APT are not only the complex preparation routes, but also the rather complex nature of the field evaporation process. While single atoms typically evaporate from metallic samples, molecular ions of varying size^24–26^ are typically observed for non-metallic samples, with just a small fraction of atomic ions, sometimes arising from the dissociation of metastable molecular ions during the flight. Earlier measurements of thin water layers^17,21,27^ and recent observations from thick ice layers^15,16^ show a strong tendency in the creation of protonated water clusters of varying size. Atomic hydrogen and oxygen are rarely detected and mostly fragments from the dissociation of molecular ions^28–30^. This not only limits the spatial resolution, but also gives a higher probability of peaks from different species overlapping, hindering the detection of signals stemming from matter incorporated or dispersed in the water matrix.

In principle, APT is a calibration-free technique to determine the chemical composition by simply counting each atom, but deviations are reported for carbides^31–33^, boron^34^, nitrides (AlN,GaN)^28,30,35,36^, oxides^28,35,37^, alkali halides and other semiconducting materials^38–40^. In the case of non-metals, the composition measured by APT is not as accurate, and the specific loss of oxygen and nitrogen is commonly attributed to neutral species formed by molecular dissociations^28–30^, thereby impeding their detection. These additional uncertainties should be accounted for in interpretation, otherwise the derived conclusions can be erroneous. If such effects cannot be avoided, understanding the loss and, in the simplest cases, defining and using correction factors becomes of paramount importance. For measurements of frozen liquids or solutions, or objects dissolved in a liquid environment, standards and possible corrections are not yet available.

The importance of water in biological processes is undisputable. Most biochemical processes are enabled by the unique properties of water as a polar medium. Central examples of biologically relevant molecules are carbohydrates, glucose being one of the simplest ones. They are crucial for the energy balance and integral to most important biological information storage, RNA and DNA^41^. Glucose is a monosaccharide consisting of a pyranose ring, which contains five carbon atoms and one oxygen atom, with a hydroxymethyl group at C5. The molecular formula for glucose is C_6_O_6_H_12_. In its solid form, the ring is usually closed, while in solution a small fraction of the molecules transforms into an open chain. The most dominant representations of this type are α- and β-pyranose^42^. Its molar mass accounts to 180.16 g mol^−1^ and the maximum solubility in water is 470 g l^−1^ at room temperature. This equates to a solubility of 2.608 mol l^−1^ or, on an atomic scale, 2 glucose molecules per 42 water molecules (x_S_ = 1/22).

Equipped with the background knowledge from the prior analysis of pure Mili-Q water^15^, we focus in this study on the differences in the field evaporation behaviour between a saturated glucose solution, bulk glucose and pure water to broaden the discussion basis and advance the understanding and map the possibilities as well as limitations inherent to the application of state-of-the-art cryo-APT to the analysis of solutions, molecules and biological materials.

## Results and discussion

Through laser assisted APT, we acquired data from 7 specimens, yielding at least 30 million events each. Stable measurement conditions, with a homogeneous detector pattern and a steady smoothly increasing voltage curve over time, were obtained with a detection rate of 3 ions per 1000 laser pulses, at a repetition rate of 100 kHz and a pulse energy between 40 and 80 nJ. Chemical information is derived from the measured time of flight (ToF). The ToF measurement is continuously restarted by the emission of a new laser pulse, defining the maximum opening time of the ToF measurement window. While the maximum detectable mass for a flight length of 120 mm at 6 kV and a repetition rate of 200 kHz accounts to 200 u, the range can be extended at 100 kHz to about 800 u. Such large masses are normally of limited interest for APT, but at 100 kHz we could detect significant signals stemming from large molecules up to a mass of 350 u. For high laser repetition rates these large fragments will have not enough time to reach the detector before a new laser pulse is initiated. Besides, the decreased repetition rate enables samples with low thermal conductivity to cool down between laser pulses manifesting in an improved signal to noise ratio^16^.

**Figure 1:**
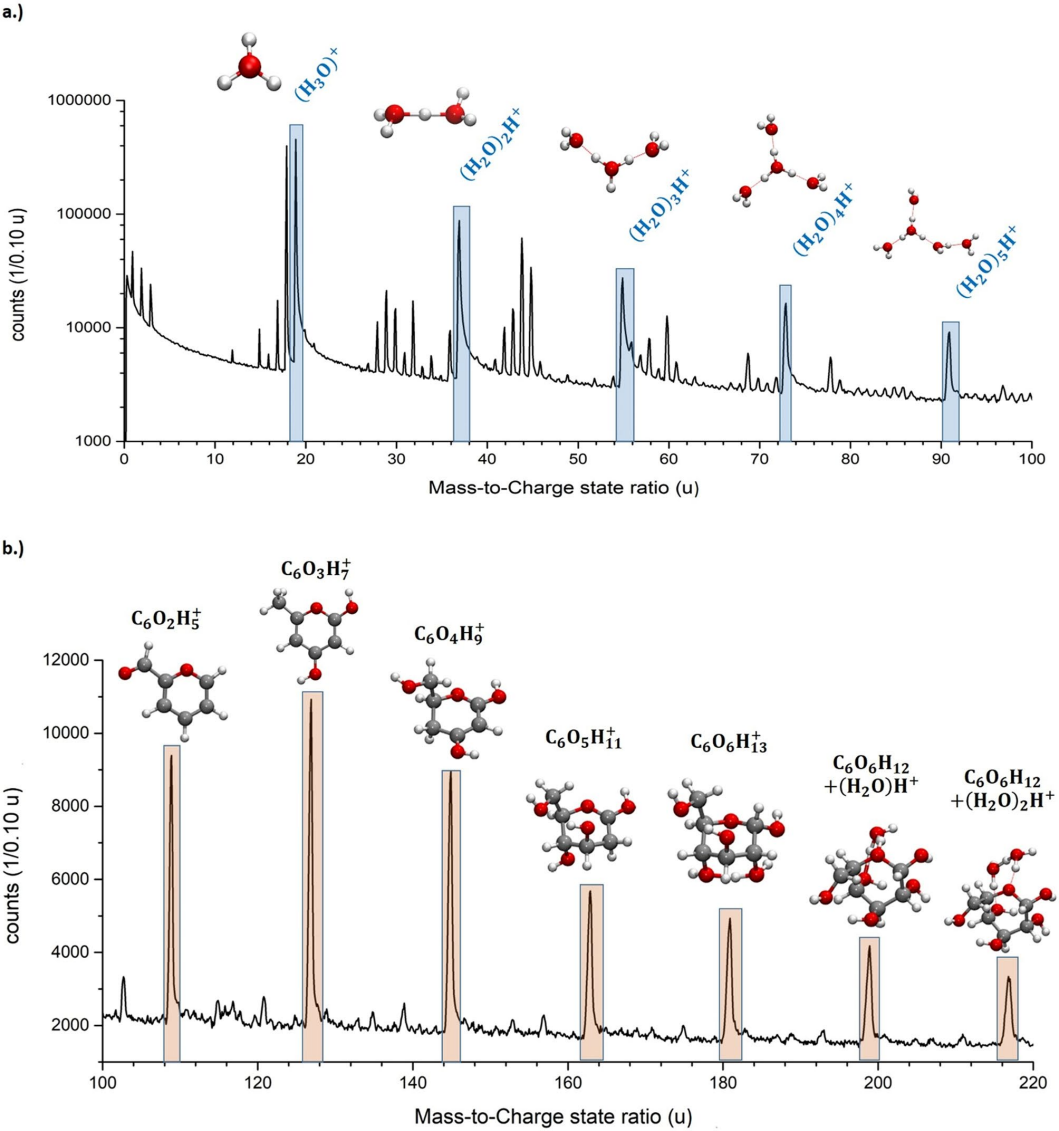
(**a**) Mass spectrum of a supersaturated glucose solution with a mass-to-charge state ratio from 0 to 100 u as logarithmic plot. The protonated water molecules/clusters are highlighted and the respective molecular structure of (H_2_O)_*n*_H^+^ with *n* = 1–5 are shown. (**b**) Mass spectrum of a supersaturated glucose solution with a mass-to-charge state ratio from 100 to 220 u as linear plot. The peaks stemming from glucose from m/z = 91 up to 217 u are marked and the respective molecular structures are shown.

The mass spectrum of the saturated glucose solution (Fig. 1a,b) reveals more than 140 mass peaks, a clear increase compared to the pure water spectra^15^. Peak identification is ambiguous due to the large number of possible combinations of charge states and their elemental combinations. We generally favoured the simplest explanation for a specific mass signal, using the lowest possible charge state option to assign a molecular identity to each signal, since the effective evaporation field for water is low^15,16^ and thus, lower charge states are more likely to be observed. Different approaches to assign individual peaks will be discussed below and compared to the expected ratio of water to glucose molecules. The first 10 million atoms were excluded from the evaluation, in order to preclude preparation artefacts stemming from the annual milling process.

### Mass spectra comparison of glucose solution to pure water

**Figure 2:**
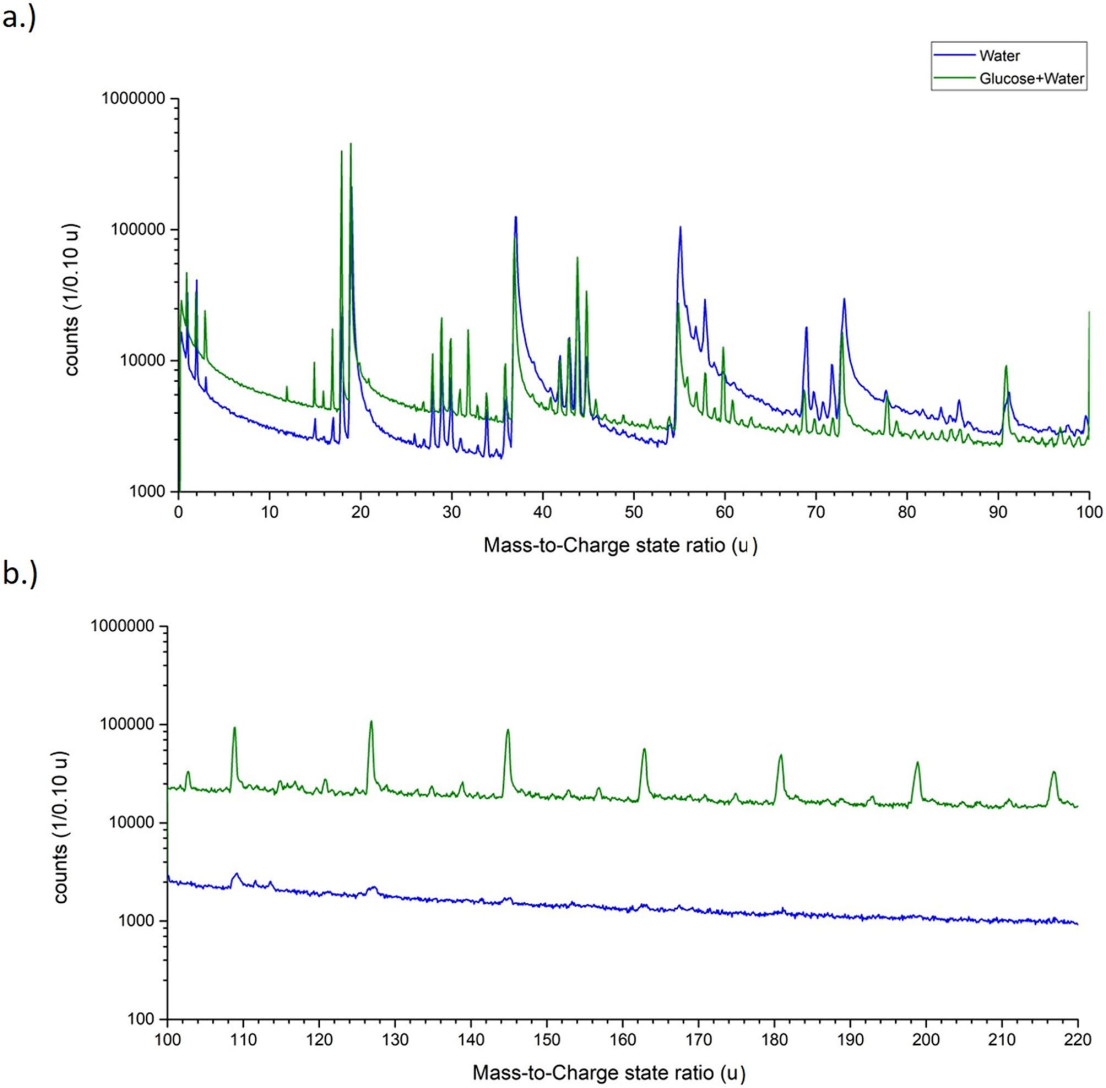
Comparison of the mass spectra of pure water and a saturated glucose solution. In (**a**) from the mass-to-charge state ratio of m/z = 0–100 u and in (**b**) from m/z = 100–220 u as logarithmic plot. In (**b**) the mass spectrum of the supersaturated glucose solution was shifted upwards by a factor of 10 to make both spectra more visible.

Since water is the majority compound in the saturated glucose solution (x_S_ = 1/22, 470 g L^−1^ at 20 °C), it is reasonable to expect a mass spectrum with significant similarity to pure water^15^. We observe the same protonated water-containing ions (H_2_O)_*n*_H^+^ with *n* = 1–5, with peaks at m/z = 19, 37, 55, 73 and 91 u, as for pure water(Fig. 2a). The peak shapes of the signals match to the previous measurement of water with regard to their peak width, relative intensity and the pronounced tailing^15^. Furthermore, higher charged signals corresponding to the composition (H_2_O)_*n*_(OH)_*m*_^2+^ and their satellite peaks (Δm =  + 1) match as well. Distinct differences become visible for larger atomic masses. Periodic signals at masses larger than m/z = 100 u are readily visible in Figs. 1b and 2b. These high-mass signals have a distinct mass difference of 18 u. All signals stemming from m/z ratios above 91 u could, in principle, be explained by large protonated water clusters (H_2_O)_*n*_H^+^ with *n* > 5, which, however, are not observed in this quantity in earlier measurements of pure water (Fig. 2b).

**Figure 3:**
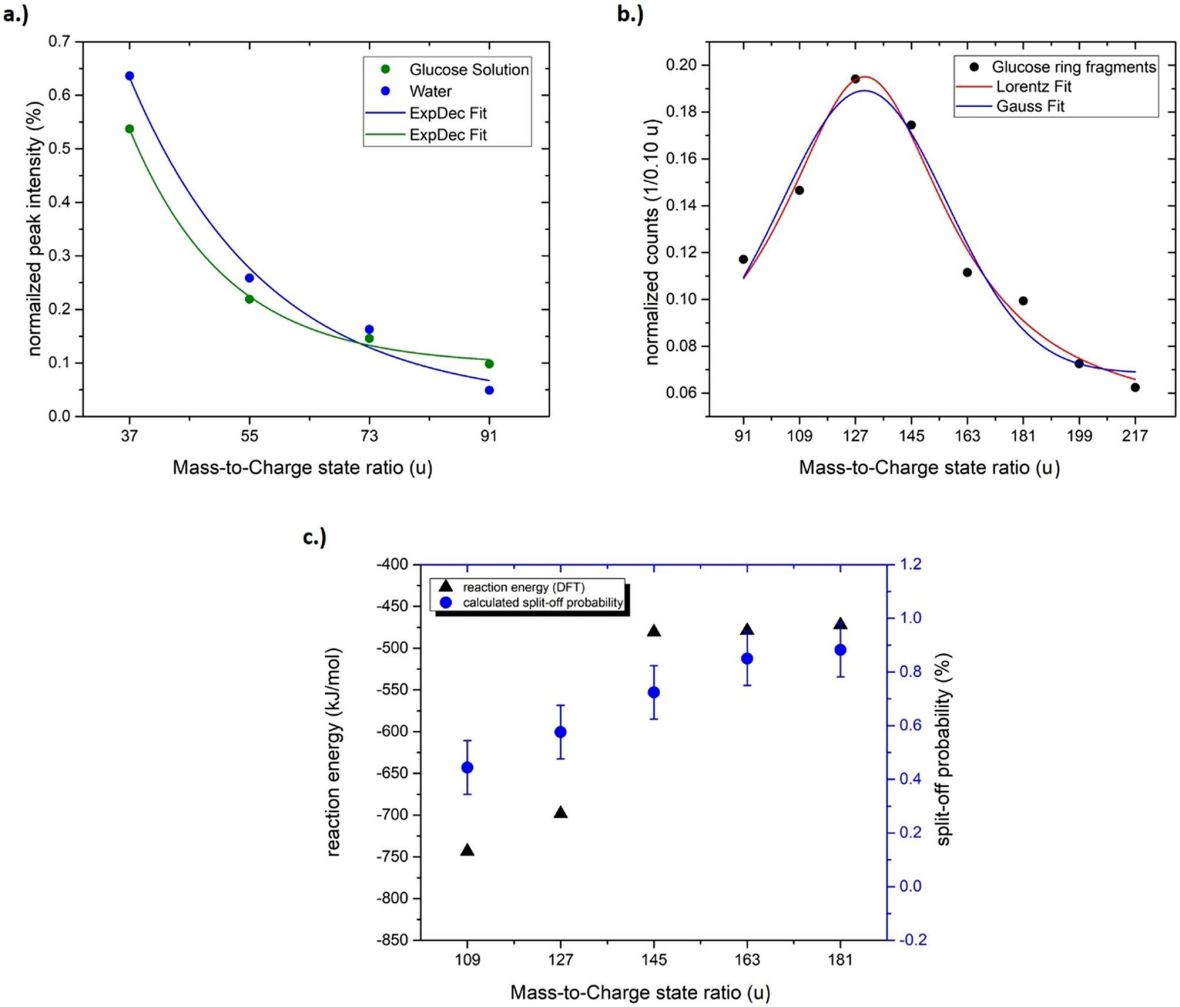
In (**a**) the relative peak intensity of the water molecules (H_2_O)_*n*_H^+^ with *n* = 1–5 from a pure water measurement and the glucose solution, in (**b**) the peak distribution of the peaks of the glucose ring from m/z = 91 up to 217 u and in (**c**) the calculated probabilities (Table 2) and the reaction energy calculated by DFT to remove one HO + H from a glucose ring-/fragment are plotted against the respective mass-to-charge state ratio.

The glucose molecule has a mass of 180 u. A reasonably strong signal appears at m/z = 181 u and can be also interpreted as a protonated glucose molecule. Glucose contains seven hydrogen atoms and five OH-groups attached to different carbon atoms. The periodic peaks with lower masses than 181 u can be explained by the simultaneous removal of an OH group and one H-atom from the carbon atoms forming the pyranose ring. The removal of (HO + H)_*n*_ with *n* = 0 – 5 would result in the distinct spacing of mass peaks less than 181 u with a mass difference of Δm/z = 18 u (H_2_O^+^). This periodicity stops once all OH groups are removed (Fig. 2b), at m/z = 91 u. To complete the chain of evidence, relative peak intensities are determined for protonated molecules (Fig. 3a). The relative intensities of the water cluster signal at m/z = 37, 55, 73, and 91 u agree with the intensity distribution determined for pure water, implying a similar effective evaporation field strength for both liquids. Beyond m/z = 91 u the relative intensities increase again in the case of glucose solution (m/z = 91, 109, 127, 145, 163, 181, u). Since measurement conditions are comparable and such large clusters of water molecules were absent in the case of pure water, all signals with high masses above 91 u are therefore attributed to glucose.

### Molecular ions consideration

**Figure 4:**
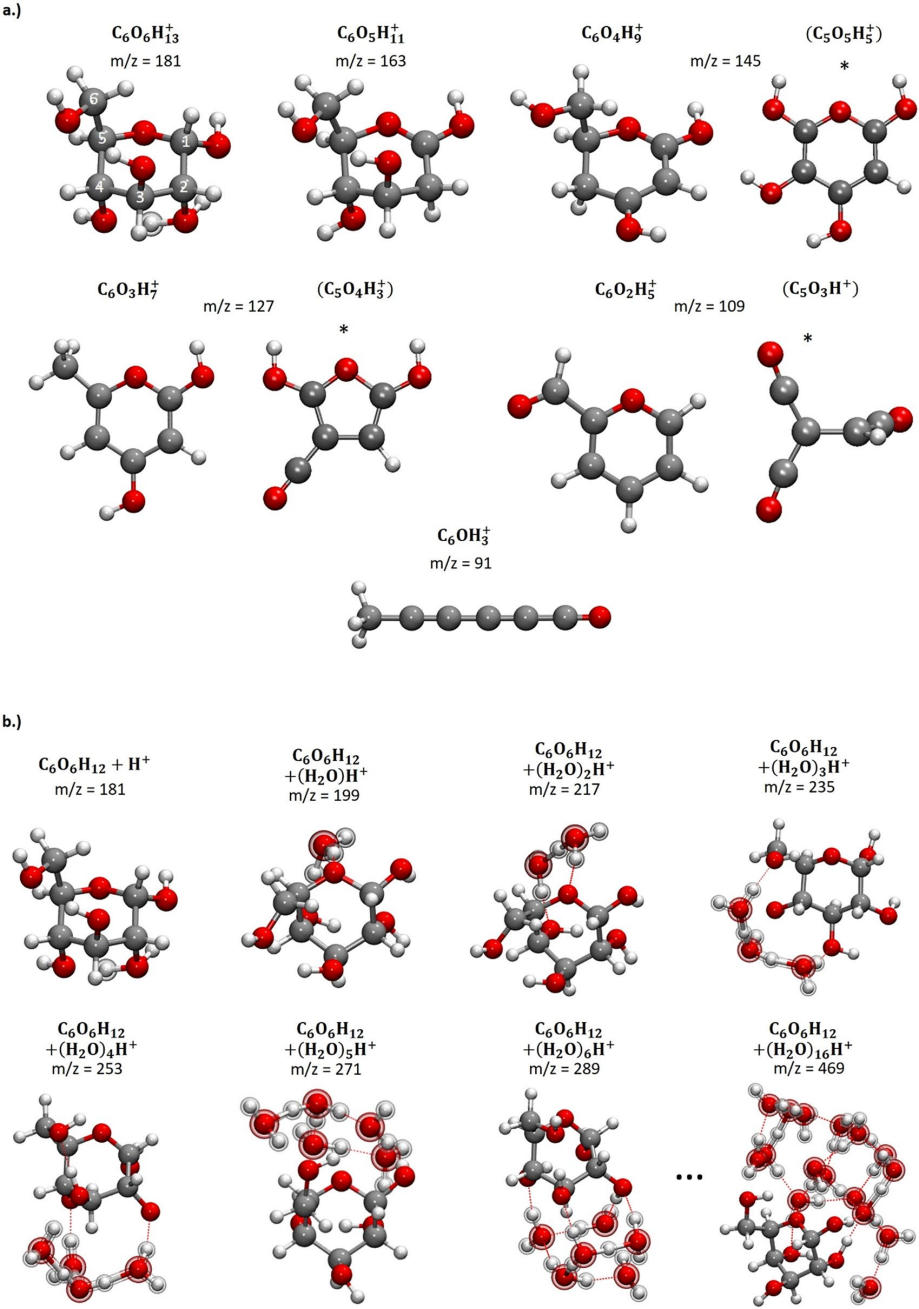
(**a**) Molecular structures corresponding to the mass-to-charge state ratios of m/z = 181–n∙18 u with *n* = 0 to 5 until m/z = 91 u. All structures shown here represent the isomers with the lowest energy. Alternative molecules at m/z = 145, 127 and 109 u are marked with (*). However, they are deemed unlikely, because they would require the simultaneous removal of one carbon along with several H atoms. (**b**) Molecular clusters formed by adding cations to glucose: left to right: glucose molecule (α-D-glucopyranose) with addition of H^+^, H_3_O^+^ and H_5_O_2_^+^ until (H_2_O)_6_H^+^, which form water clusters bound to glucose via hydrogen bonds (dotted lines). The structure shown for C_6_O_6_H_12_ + (H_2_O)_16_H^+^ is the result of an MD simulation with GFN-xTB, followed by an energy minimization with BP86/def2-SVP. It is obvious that the glucose molecule locates on the surface of a water cluster. The additional atoms are emphasized by depicting them in a different style.

DFT calculations have been performed to calculate the structure and the stability of the formed glucose fragments. The molecular ions smaller than the glucose molecule are depicted in Fig. 4a. In each case, the equivalent of *n* water molecules *n* = 1—5 has been removed compared to the structure at m/z = 181 u. At the mass-to-charge state ratios of m/z = 127 u and m/z = 109 u, isomers with planar 6-membered rings were found. These 6-membered pyrylium rings are aromatic, which renders these isomers particularly stable. The last molecule at m/z = 91 u has a linear structure.

After removal of OH + H, the remainder of the glucose molecule undergoes a molecular rearrangement. Due to their inherent mobility, the remaining hydrogen atoms relocate and bind to a different carbon atom. In the case of C_6_O_6_H_13_^+^ (m/z = 181 u), it is possible to remove an H_2_O from one carbon atom. H_2_O is removed from C2, and subsequently one hydrogen relocates from C1 to C2, resulting in the structure shown in Fig. 4a. It is unknown which OH molecule and which hydrogen atom are removed in the smaller fragments.

Each of the structures has been found to be stable against the abstraction of the equivalence of one H_2_O from the ring structure except for the clusters at m/z = 181 u and m/z = 163 u , which are therefore metastable. However, in those cases, the calculated dissociation energy is rather small, 1.5 kJ mol^−1^ for m/z = 181 u and 8 kJ mol^−1^ for m/z = 163 u (Table 1).

**Table 1:** Calculated reaction energies for H_2_O and OH + H addition to selected glucose fragments.

m/z of the product	Δ*E* with respect to H_2_O		Δ*E* with respect to OH + H	
	(kJ mol^−1^)	(eV)	(kJmol^−1^)	(eV)
109	− 263	− 2.73	− 744	− 7.71
127	− 218	− 2.26	− 698	− 7.23
145	0	0	− 481	− 4.99
163	2	0.02	− 479	− 4.96
181	8	0.08	− 472	− 4.89
199	− 90	− 0.93	− 571	− 5.92
217	− 107	− 1.11	− 588	− 6.09

Molecular structures with alternative compositions were found for m/z = 145, 127 and 109 u (see Fig. 4a marked with *). In contrast to the structures shown in Fig. 4a,b, they contain only 5 instead of 6 carbon atoms. The formation of these structures is, however, considered unlikely as it would require a fragmentation of the carbon skeleton of the molecule, followed by the simultaneous removal of one carbon, one oxygen and several hydrogen atoms. A direct comparison of the energy to those structures shown in Fig. 4b is impossible due to the different composition.

The reaction energies listed in Table 1 are calculated using the following equation:

1 $$\Delta E\left( {m/z} \right) = E\left( {m/z} \right) - E\left( {m/z - 18} \right) - E\left( n \right)$$

where *E* denotes the electronic energy + zero-point correction from the DFT calculations and E(n) refers to either E(H_2_O) or E(H + OH), respectively.

Molecules with a mass-to-charge state ratio higher than m/z = 181 u, following with a Δm/z = 18 u, are also detected in considerable quantity and most likely correspond to a glucose molecule with one or more additional cations (H^+^, H_3_O^+^ and H_5_O_2_^+^, depicted in Fig. 4b) bound via hydrogen bonds to the glucose ring.

An analysis of the partial charges shows how the charges are distributed within the cluster. The positive charge is delocalized over the whole cluster for m/z = 163 u and below, while a clear charge localization can be observed for clusters of glucose and water. In the cluster with m/z = 199 u, i.e., a hydronium ion attached to glucose, the positive charge is mainly localized at the hydronium ion. At m/z = 217 u, 79% of the overall positive charge is localized at H_5_O_2_^+^, the Zundel ion. Most of the molecule’s positive charge is therefore located in the water component of the cluster and this portion increases with the cluster size. For the biggest cluster depicted in Fig. 4b, the percentage increases to 97%.

In a saturated glucose solution, there are 21 water molecules available to solvate each glucose molecule. Further molecules at higher mass-to-charge ratio with more than two additional water molecules with the formula C_6_O_6_H_12_ + (H_2_O)_*n*_H^+^ with *n* = 2 – 9, could also be observed (see supplementary material Fig. A 5). The respective stabilities and the arrangement of larger water clusters around the glucose molecule were also computed. The DFT calculations show that it is energetically favourable to build compact water clusters bound via hydrogen bonds to the glucose molecule instead of arranging water molecules around the glucose molecule as a hydration shell (Fig. 4b).

The apparent distribution of relative intensities above m/z = 91 u, assumed to stem from glucose molecules show a maximum centred at m/z = 127 u (Fig. 3b). From the analysis of multi-hit events^35^ on the detector, no preferential co-evaporation of HO, H_2_O, H_3_O with a large glucose cluster could be observed (see supplementary part Fig. A 1–A 4). Therefore, the loss of HO + H from the glucose molecule does obviously not occur after evaporation by dissociation. Either the HO + H fragments stay behind inside the water matrix, after the major carbon ring has been evaporated, or they are ripped of solely before evaporation of the main ring. However, the observed intensity distribution cannot be statistically modelled if the same probability is assumed for the separation of each water fragment.

To explain this, let us perform a thought experiment. We imagine the glucose molecules statistically distributed within a matrix. Like raisins in a cake. This cake is slowly eroded from the surface. Whenever a raisin comes to the surface, we try to pick it. If we succeed, we wait for a next one. If we do not succeed, at least a small defined fragment of the raisin tears off, while the rest remains in the cake. We then have another try to pick the remaining raisin or grasping at least a fragment. We are not supposed to care about the time between attempts. So, it is sufficient to consider the relative probabilities for picking the raisin, meaning the carbon ring p_evap_(k) that has lost k-1 (HO + H) molecules before or for catching the next small fragment, meaning the kth (HO + O) molecule p_split_(k) = 1-p_evap_(k).

A glucose ring having lost k-1 (HO + H) molecules can only be observed if before k-1 splitting attempts have been successful. Thus, the probability to observe such ring fragment, which corresponds to the relative abundances in the mass spectrum reads

2 $$\frac{{n^{k} }}{{\mathop \sum \nolimits_{i} n^{i} }} = p_{Fragment} \left( k \right) = p_{evap} \left( k \right) \cdot \mathop \prod \limits_{n = 1}^{k - 1} p_{Split} \left( n \right) \quad with\;k\epsilon \left\{ {1 \ldots 6} \right\}.$$

In turn, the splitting probabilityof the kth (HO + H) molecule can be calculated

3 $$p_{Split} \left( k \right) = 1 - {{p_{Fragment} \left( k \right)} \mathord{\left/ {\vphantom {{p_{Fragment} \left( k \right)} {\mathop \prod \limits_{n = 1}^{k - 1} \left( {1 - p_{Evap} \left( n \right)} \right)}}} \right. \kern-\nulldelimiterspace} {\mathop \prod \limits_{n = 1}^{k - 1} \left( {1 - p_{Evap} \left( n \right)} \right)}}\quad with\;k\epsilon \left\{ {2,3,4,5.6} \right\}.$$

To match the observed frequency distribution with this simple concept, the relative probability for evaporation of the carbon ring has to increase continuously after each removal of an HO + H fragment (k > 1). Or in other words, the further removal of another HO + H event becomes harder (see Table 1).

Being a Gedankenexperiment, this simplified approach has limitations, but the decrease in split-off-probability is supported by DFT calculations and correlates with experiments (see Fig. 3c), since the remaining fragment become more stable against further dissociation with every (HO + H) molecule removed (see Table 2). Nevertheless, a more accurate consideration has to regard the impact of the electrical field on the molecule stabilities. Once all OH groups have been released, the molecule opens into a linear structure (Fig. 4a) as this structure is energetically more stable. Fragments smaller than C6 can be explained by the splitting of the C_6_H_3_O^+^ chain.

**Table 2:** Calculated evaporation and split-off probability of the glucose molecules assuming a simple multi-step random process.

Mass-to-charge state ratio (u)	181	163	145	127	109	91
Evaporation attempt k	1	2	3	4	5	6
P_Fragment(_(k)	0.12	0.13	0.21	0.23	0.18	0.13
Evaporation probability p_evap_(k) for the complete molecule	0.12	0.15	0.28	0.43	0.58	1.00
H + OH split-off probability p_Split_(k)	0.88	0.85	0.72	0.57	0.42	0.00

Between the main peaks at m/z = 109, 127, …217 u, small peaks appear that are separated by a distance of 1 u, corresponding to a single additional hydrogen atom. There are always three very prominent peaks between two main peaks, with a distance of Δm/z = 5, 7, 12 u from the previous peak. These peaks between the mass-to-charge state ratio of m/z = 109–181 u can be associated to C_4–6_O_*x*_H_*y*_. However, the intermediate peaks in the range m/z = 181–217 u can only correspond to the molecular ions C_5_O_5_H_11-14_ + (H_2_O)_*x*_H_y_^+^ with *x* = 2–3.

The identification of an ion only by its mass, of course, does not relay anything about the internal structure of the detected species. In this specific case, the structure and the identity of the injected molecules are a priori known, but it would not be possible to differentiate common monosaccharides like galactose and fructose based on the molecular mass. On the other hand, it is unclear whether the situation would improve much if, instead of large molecules (intact or fragments) all constituents were evaporated only atomically. Omitting all aberration artefacts that may occur, the respective molecules would have to be determined from the local amount of carbon atoms for example. Detector efficiency increased constantly to about 80%-85% in most modern detectors. However, still a significant number of events is lost. Implementing problems in distinguishing a C6 ring from a C5 ring in all cases. Misplacing atoms, due to deviations in trajectories, would make the task of molecular identification from the totality of atomic signals even more difficult. This would require a more sophisticated analysis of the data, and likely statistical comparison with a database of fragments, as is done in proteomics^43^. One may speculate, whether the observed frequency distribution of fragments as described in Table 2 is unique for glucose and allows a clear distinction from other monosaccharides. Typically, the respective evaporation field scales with the sublimation energy, which is increasing with the melting temperature of the substance^2,44^. The monosaccharides at least differ in their melting temperatures (glucose 146 °C, fructose 106 °C, galactose 164 °C). In a first view, one could assume that the melting point mostly affects the probability to evaporate the complete molecule, while the probability to split OH or H in both cases covalently bond might be less influenced. Thus, the relative fractions of the high-mass peaks would change, which may result in a distinguishable pattern.

The exact identification of fragments for lower masses is even more complicated. For all lower mass peaks, combinations of different C_*x*_H_*y*_/C_*x*_O_*y*_H_*z*_ permutations in various charge state modifications can be found. A full interpretation list can be found in Table A 1 of the supplementary part.

### Comparison of a saturated glucose solution to bulk glucose

In the measurements presented before, water is the majority component. Thus, the situation is less transparent, as signals stemming from water claim the same positions within the spectrum as the sugar molecules. To avoid such superposition, we also investigated pure bulk glucose samples to identify peak positions of possible highest overlap. Still, the ionization field strength of frozen water is rather low (2–6 V/nm)^17,27,45^, while we can expect for solid sugar field strengths of some tens of V/nm, which may hinder a direct comparison.

**Figure 5:**
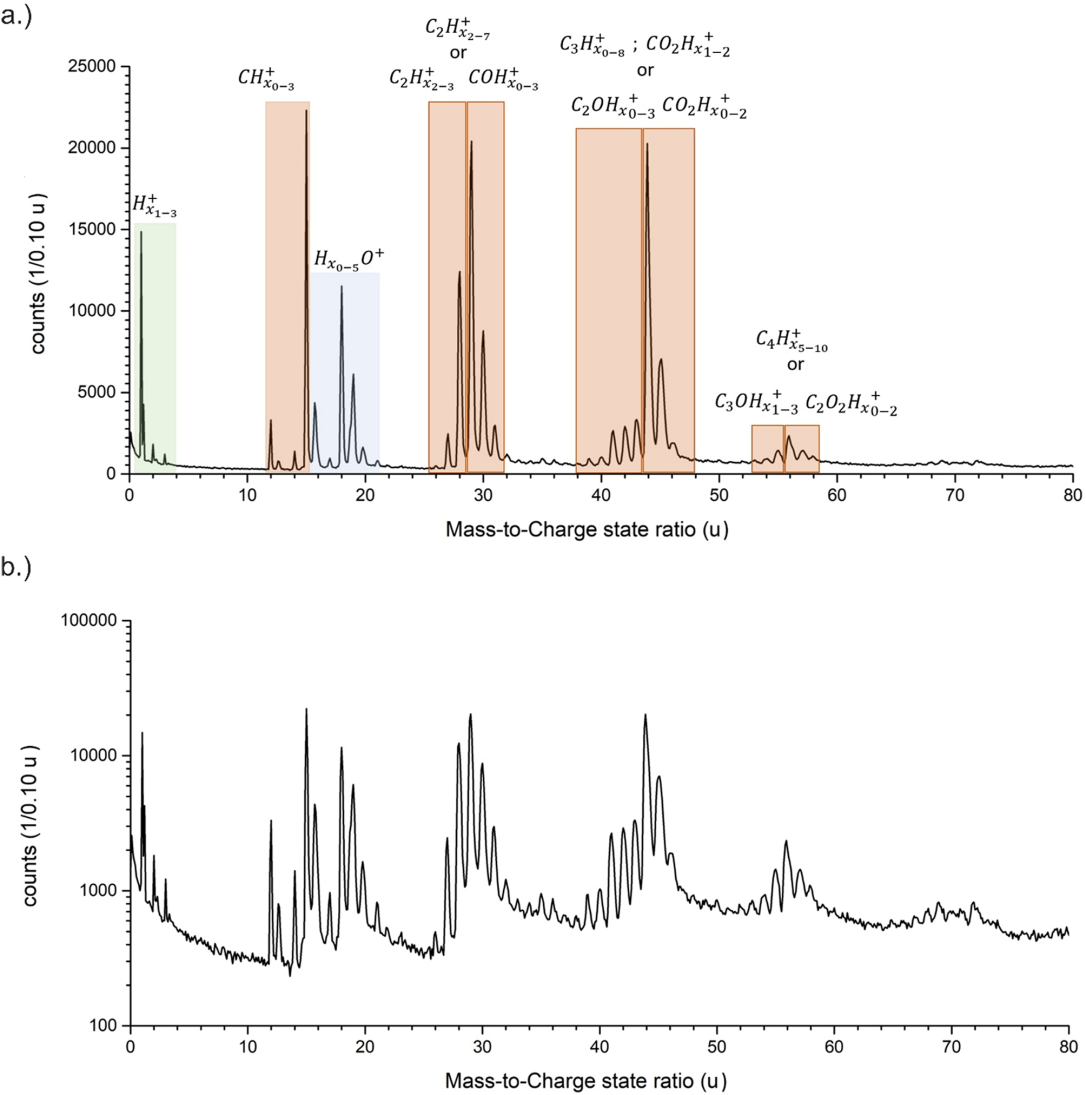
The mass spectra of the glucose bulk specimen. In (**a**) from the mass-to-charge state ratio of m/z = 0–80 u as linear in (**b**) from m/z = 0–80 u as logarithmic plot.

Samples were prepared from pure solid bulk glucose by FIB lift-out (see “Materials and methods” section)^46^. Comparing the mass spectrum of bulk glucose plotted in Fig. 5 with that of glucose in aqueous solution (Fig. 1), significant differences are readily visible. First, for bulk glucose, no signals above m/z = 80 u are detected, indicating that the average field condition is sufficient to split the pyranose rings from the glucose molecule. Focusing on lower mass signals, most peaks pertain to molecular ions, although small, but significant C (12 u), and H (1 and 2 u) atomic signals are present as reported for other carbon-based system, with varying finger prints^47–50^.

Nevertheless, series of peaks appear that are spaced by 1 u up to a mass-to-charge state ratio of m/z = 80 u. A full interpretation list can be found in Table A 2 of the supplementary part. The most intense peaks are at m/z = 15 u, corresponding to CH_3_^+^, m/z = 29 u attributed to C_2_H_5_^+^ or COH^+^, and at m/z = 44 u giving multiple identification options (C_3_H_8_^+^, C_2_OH_4_^+^ or COO^+^).

Various peaks can be explained by combinations of carbon, oxygen and hydrogen (C_*x*_O_*y*_H_*z*_). For example, in the range of m/z = 28–31 u COH_*z*_ combinations can fit. The interpretation is not univocal. Many possibilities exist for each peak (see Table A 2 supplementary part), and because of the electric field conditions, only singly charged molecules are considered. However, a comparison of all shown measurements reveals a very different fragmentation behaviour of the glucose, depending on whether it is dissolved in water or evaporated as bulk material. Bulk glucose shows a high fragmentation rate, whereas glucose dissolved in water has a lower fragmentation rate and frequently evaporates as a complete molecule. This observation must be traced back to the different bonding conditions in the solid and dissolved state and the respective electrical field strength necessary to cause field evaporation.

A further difference can be observed regarding the intensities of the detected molecules H_2_O^+^ and H_3_O^+^. In the aqueous medium, the molecule H_3_O^+^ is dominant, but in the solid state the molecule H_2_O^+^ is much more present, which could be due to a cleaving effect of the HO and H parts of the pyranose ring.

This significant difference in the ratio of H_2_O^+^ to H_3_O^+^ seems to be a reasonable marker between the discussed measurements. Generally, the mass signals stemming from the solid samples display sharper peaks and less tailing compared to the aqueous solution samples. A simple explanation by a difference in heat conductivity λ is not feasible, since the reported values for a saturated aqueous glucose solution and glucose crystals are both in the range of 0.5 ± 0.1 W m^−1^ K^−1^^51,52^, while the reported values for ice are about 5 -10 times higher, depending on the crystal structure with the lower limit given by low-density amorphous ice to be 0.6 W m^−1^ K^−1^^53^. The increased tailing has to be attributed to a more direct influence of the ice^15^.

It is insightful to discuss and interpret the correct determination of the oxygen content. The interpretation of all signals stemming from pure bulk glucose as carbohydrates C_*x*_H_*z*_ results in an extreme underestimation of oxygen (Table 3), which cannot be simply explained by the loss of neutral oxygen during the APT measurement.

**Table 3:** Calculated stoichiometry by different peak interpretations.

	C_*x*_/H_*y*_	C_*x*_O_*y*_H_*z*_	C_*x*_H_*y*_/C_*x*_O_*y*_H_*z*_ 0.5/0.5	C_*x*_H_*y*_/C_*x*_O_*y*_H_*z*_ 0.25/0.75	C_*x*_H_*y*_/C_*x*_O_*y*_H_*z*_ 0.2/0.8	C_*x*_H_y_/C_*x*_O_*y*_H_*z*_ 0.1/0.9
Oxygen loss	86.25%	0	50.29%	23%	15.66%	0.13%
Oxygen excess	0	18.31%	0	0	0	0
Hydrogen loss	0	32.90%	0	8.16%	12.33%	21.73%
Hydrogen excess	31.2%	0	8.65%	0	0	0

The overlap of C_x_H_y_ and C_x_O_y_H_z_ in certain areas were calculated (see supplementary part Table A 2 marked in red).

Instead, one may favour the interpretation of the mass signals as C_*x*_O_*y*_H_*z*_ molecules. In this case, the amount of available oxygen is overestimated by 18.31%. Thus, the reality seems to be a convolution between both possibilities as suggested in the table. Further experiments are needed to resolve the sources of uncertainty.

### Determination of compositional information for the aqueous solution

In general, compositional information is of tremendous interest. Identification of compositions, and phases thereof, diffusion coefficients, and segregations factors are important cornerstones to identify processes and underlying mechanisms. In our case, the initial composition of the solution is well known. Since glucose and other sugars act as cryoprotectants and form glasses for highly concentrated solutions^54^, it can be used to judge the peak identification and to identify open questions and problems to be addressed in the future.

A bijective identification of the retrieved mass information is difficult due to the already discussed overlapping mass signals and the large number of possible explanations. Various combinations of (H_2_O)_*n*_H, C_*x*_H_*y*_, C_*x*_O_*y*_H_*z*_ molecules would deliver reasonable explanations. Although, a combination of all sources is highly likely and increases the complexity as well as leads to uncertainty and loss of local spatial information.

For stoichiometry calculations, all peaks are identified, and the resulting number of individual events counted. Different interpretation approaches are used, and the unfolding molecule was split into its atomic components carbon (C), oxygen (O) and hydrogen (H). In addition, a baseline correction was performed to obtain an accurate determination of the atomic ratios.

All obtained carbon atoms are assumed to be originating from glucose molecules. By dividing the total number of carbon atoms by a factor of six, the total amount of glucose molecules is calculated. In a next step, the total number of glucose molecules is multiplied by six and subtracted from the total number of oxygen atoms. The remaining oxygen atoms are assumed to stem from water molecules. The saturated glucose solution should exhibit a mass mixing ration r_M_ = 0.47 of glucose to water, which corresponds to 21.27 water molecules per glucose molecule for a saturated solution (molar fraction x_S_ = 0.0449 ≈ 2/42). For the calculation of the stoichiometry different peak interpretation approaches were used (Table A 3, A 4 supplementary part) and the results are listed in Table 4.

**Table 4:** Calculated stoichiometry by different peak interpretation approaches.

Interpretation	Mass mixing ratio of glucose to water	Water molecules per glucose molecule	Hydrogen (excess) (%)
Theoretical value	0.47	21.27	0
(C_*x*_H_y_)	519.94	0.02	33.37
(C_*x*_H_*y*_) + water peaks	1.40	7.17	25.15
(C_*x*_O_*y*_H_*z*_) + water peaks	0.61	16.36	4.28
(C_*x*_O_*y*_H_*z*_) + water peaks + overlap (C_*x*_O_*y*_H_*z*_)/H_*x*_O_*y*_ − 25%/75% +	0.46	21.68	4.60
(C_*x*_O_*y*_H_*z*_) + water peaks + overlap (C_*x*_O_*y*_H_*z*_)/H_*x*_O_*y*_ − 33%/67%	0.48	20.81	4.60
(C_*x*_O_*y*_H_*z*_) + water peaks + overlap (C_*x*_O_*y*_H_*z*_)/H_*x*_O_*y*_ − 50%/50%	0.52	19.10	4.49
(C_*x*_O_*y*_H_*z*_) + water peaks + overlap (C_*x*_O_*y*_H_*z*_)/H_*x*_O_*y*_ − 67%/33%	0.57	17.47	4.41
(C_*x*_O_*y*_H_*z*_) + water peaks + overlap (C_*x*_O_*y*_H_*z*_)/H_*x*_O_*y*_ − 75%/25%	0.60	16.74	4.23

Interpreting the signals at m/z = 17, 18 and 19 u as HO, H_2_O and H_3_O, respectively, and all other peaks as molecules by various combinations of C_*x*_H_*y*_ results in a glucose to water mass-ratio of 519.94. The ratio deviates massively from the theoretical value by a factor of 1100.

Using our earlier measurements of pure water^15^ as a footprint, all peaks visible in the water spectrum are attributed to water, while large molecules are assumed to be large glucose molecules. Signals at masses below 91 u, which were not visible in the water measurements, are assumed to be hydrocarbons with the formula C_*x*_H_*y*_. The mass-ratio of glucose to water is calculated to be 1.40. In this case, it corresponds to 7.17 water molecules per glucose molecule. The ratio still deviates from the theoretical value by a factor of 3. However, it shows that the ratio has improved by a factor of 366 with only the pure re-interpretation of the water molecules. Nevertheless, there is also an underestimation of oxygen, which is caused by the overlapping of peaks or by wrong interpretation of the carbon chains.

As already mentioned, various signals can also be explained by C_*x*_O_*y*_H_*z*_. Using this interpretation only for signals not stemming from water, leads to a ratio of 0.61, which deviates by a factor of only 1.3 from the theoretical value of 0.47. This corresponds to 16.36 water molecules for each glucose molecule.

Since we observe the evaporation of large and mostly intact glucose rings, it seems unlikely from previous considerations and DFT calculations, that the pyranose ring is cracked and fragments of C_*x*_O_*y*_H_*z*_ are observed. However, DFT calculation for the fragment with mass m/z = 91 u reveal a chain-like structure with unsaturated carbon atoms. The bond energy between a C–O bond^55^ is slightly higher than that of a C–C bond^55^. H_*x*_O molecules of water can possibly recombine with the adjacent glucose fragment and therefore, C_*x*_O_*y*_H_*z*_ fragments would allow to explain the observed mass signals.

To achieve a result close to the correct stoichiometry, the results from bulk glucose (Fig. 5) are considered. Especially in the mass range from m/z = 27 to 36 u and m/z = 41 to 48 u, significant overlap with water signals occurs. By assigning 33% of the signals to C_x_O_Y_H_z_ fragments and 67% to water clusters, the mass-ratio of 0.48 is achieved. This is very close to the theoretical value, but demonstrates the ambiguity of the correct determination of the stoichiometry at the moment.

The excess of hydrogen is reduced by a factor of 1.3 only if the reinterpretation of the protonated water clusters was considered. However, the hydrogen content of still 25.15% is clearly too high, which is due to a wrong interpretation. Only with the assumption of C_*x*_O_*y*_H_*z*_ molecules and protonated water cluster is the proportion reduced by a factor of 5.2 and is about 4.28%. This overestimation of hydrogen can be attributed to residual hydrogen from the stainless-steel chamber and cannot be quantified exactly, but it is a very likely reason for the overestimation of the hydrogen content in APT measurements.

Correlative information sources have to be used to support the APT analysis.

### Three-dimensional structural analysis

**Figure 6:**
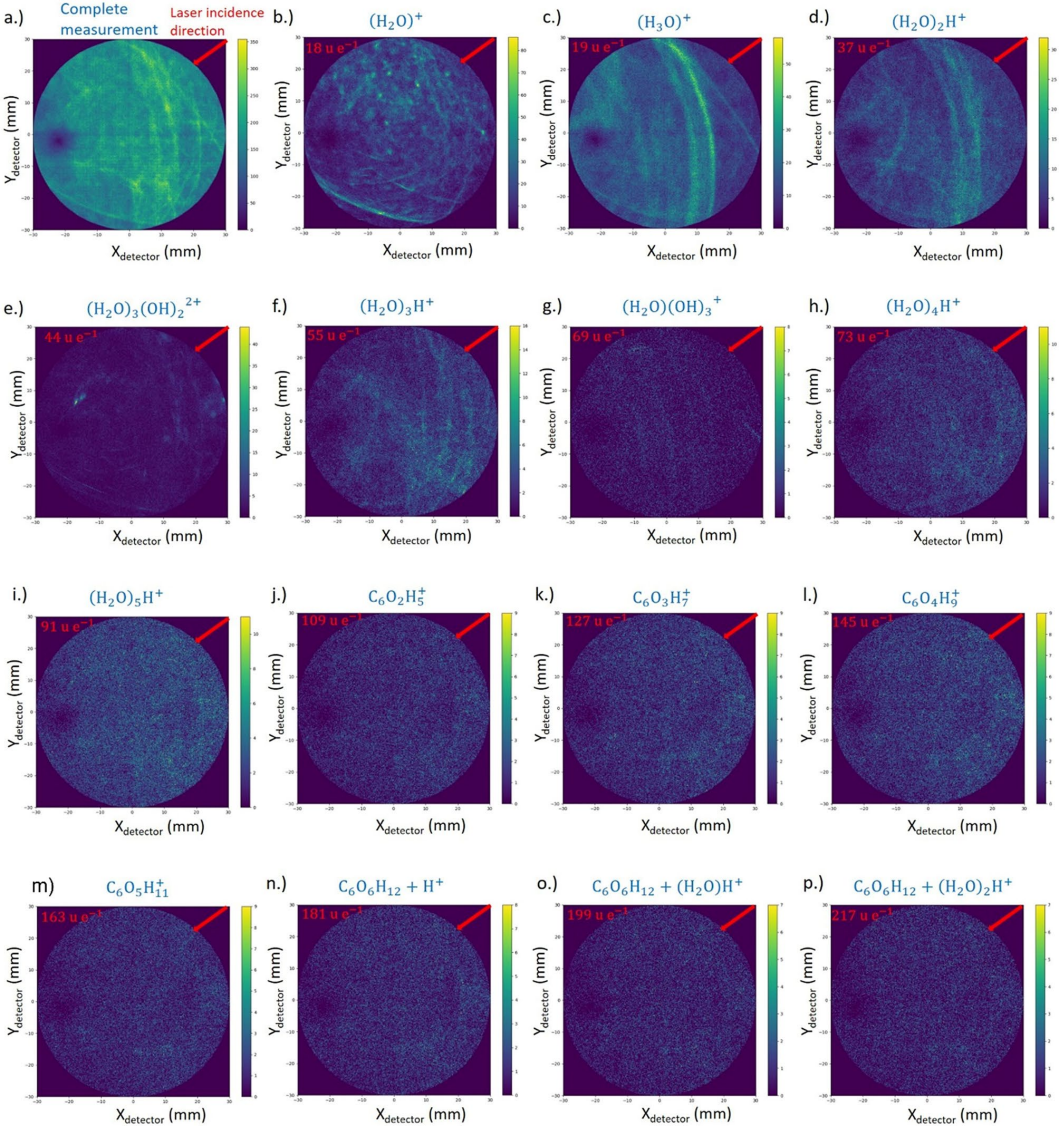
Desorption maps; (**a**) all events (**b**–**p**) desorption maps of different protonated water and glucose fragments ordered by increasing masses and size. Laser incidence direction is marked with a red arrow.

The unique feature of APT is the reconstruction of the measured volume in 3D with a near atomic resolution. With the dominating evaporation of molecular species, the principal resolution limit is controlled by the size of the individual fragments. Desorption maps for selected molecules are depicted in Fig. 6. While the distribution is homogenous especially for larger protonated water clusters and glucose molecules, local density changes for H_3_O^+^ molecules are apparent. The occurrence of line features seems to be related to the laser incidence direction, as reported for pure water^15^.

**Figure 7:**
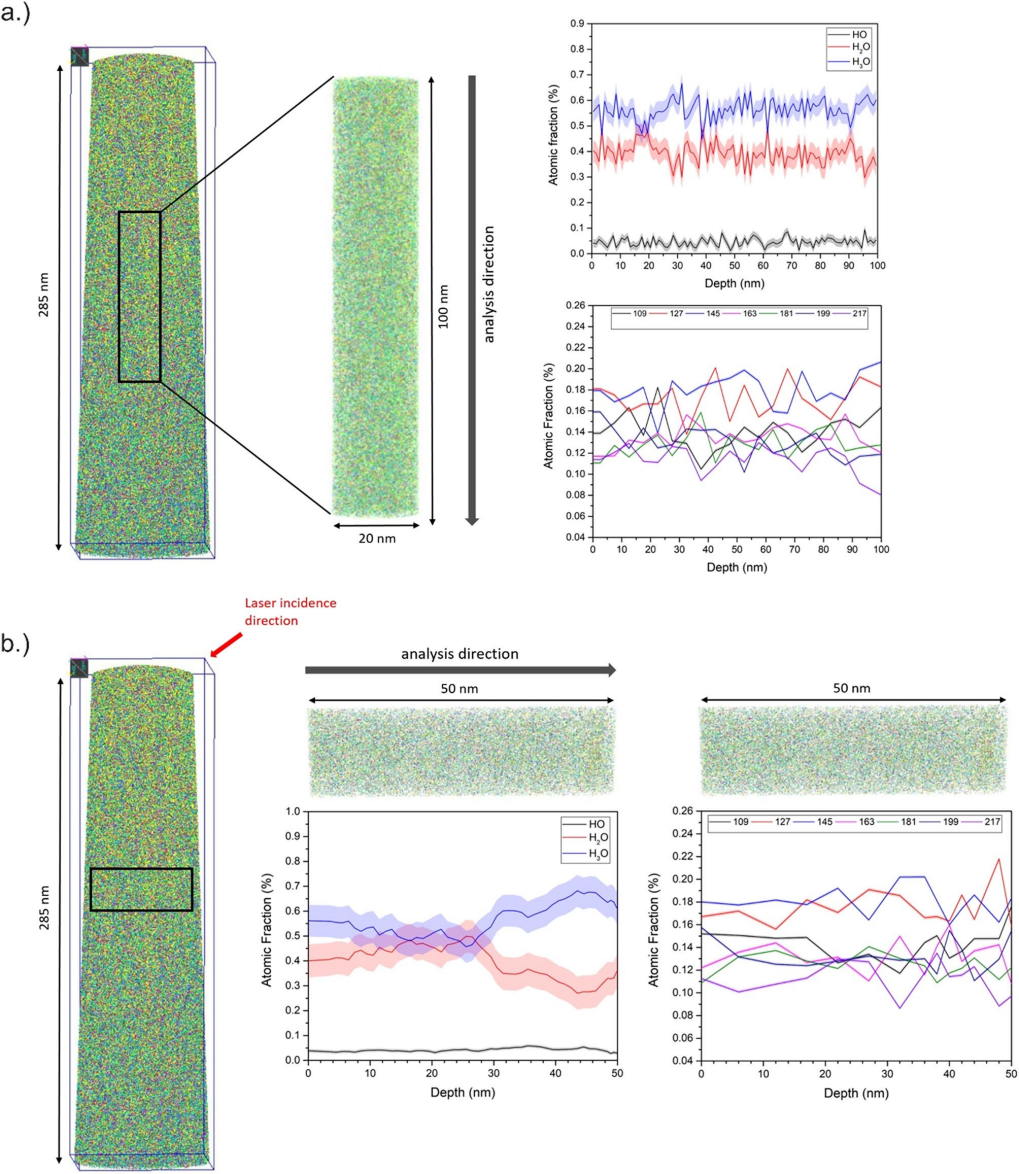
(**a**) Representative 3D reconstruction of a measured specimen with a total length of 285 nm. A cylinder with a dimension of 50 × 50 × 100 nm was selected vertical and in (**b**) horizontal to the tip from the total volume in order to analyse the composition profile of the H_*x*_O molecules with *x* = 1–3 and the glucose fragments from m/z = 91 until 217 u.

In order to investigate the distribution of the solvated glucose molecules inside the water matrix, the measurement data are reconstructed (see “Materials and methods” section for more details) (Fig. 7). As major component, the signal from water and related water clusters is dominating. Small local density imperfections become visible, which are related to the line structures in the desorption maps. Their origin must be further investigated.

The distribution of HO, H_2_O and H_3_O molecules is probed by a cylinder, dimension of 20 × 100 nm, along the tip axis (Fig. 7a) and perpendicular to the axis (10 × 50 nm) (Fig. 7b). While the ratio of the water signals is relatively constant in tip direction, a change of relative abundance from H_3_O^+^ to H_2_O^+^ is recorded perpendicular to the tip axis. Since asymmetric with respect to the tip symmetry axis, again, the laser incidence direction can be made responsible. A suggested explanation would refer to the local heating of the sample due to the laser matter interaction leading to local change in tip curvature. As a result, the effective field at the impact side of the laser is usually less than at the opposite. A lower field, appears to favour H_2_O molecules evaporating.

**Figure 8:**
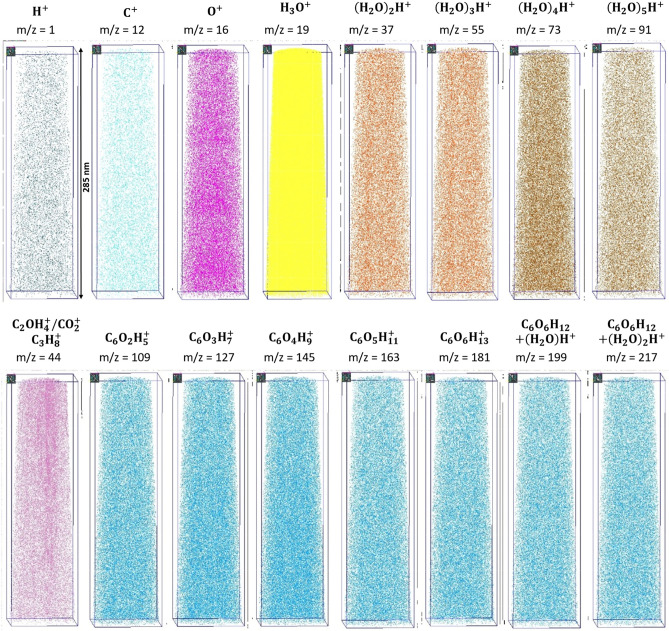
Individual maps of the reconstructed volume for different molecules are shown.

The glucose fragments show a homogeneous distribution in the 3D volume, as observed in the desorption maps, which can be attributed to a very fast cooling of the sample. Therefore, no segregation zone is observed caused by crystallization of the water (Fig. 8).

**Figure 9:**
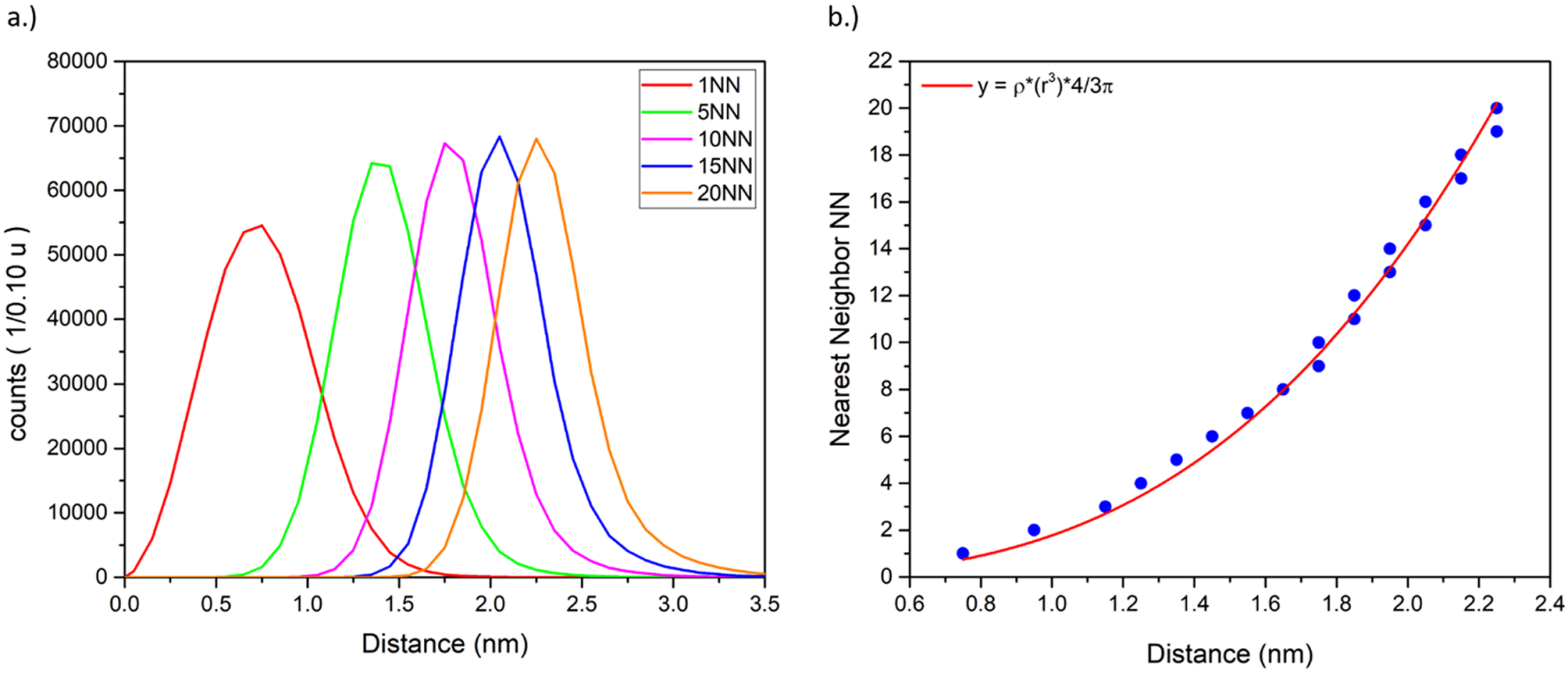
(**a**) Radius distribution to include the NN other glucose molecules around a glucose molecule. (**b**) Increase of the mean radius with number of nearest neighbours.

By determining the radius of a sphere around an identified glucose molecule (masses m/z = 109–181 u) that includes a given number of nearest neighbouring (NN) glucose molecules, the distribution of the glucose inside the solution can be investigated. The number of included nearest neighbours’ scales with the radius to the power 3

4 $$NN_{sphere} \left( r \right) = \rho \cdot \frac{4}{3}\pi r^{3}$$

here *ρ* denotes the glucose density and is determined to 0.5 ± 0.1 molecules nm^−3^. As initially described, about 1.57 glucose molecules are expected per cubic nanometre. Taking the detection efficiency of 50% into account a partial fragmentation of a distinct number of molecules into smaller fragments, the value represents quite well the expected density distribution (Fig. 9).

**Figure 10:**
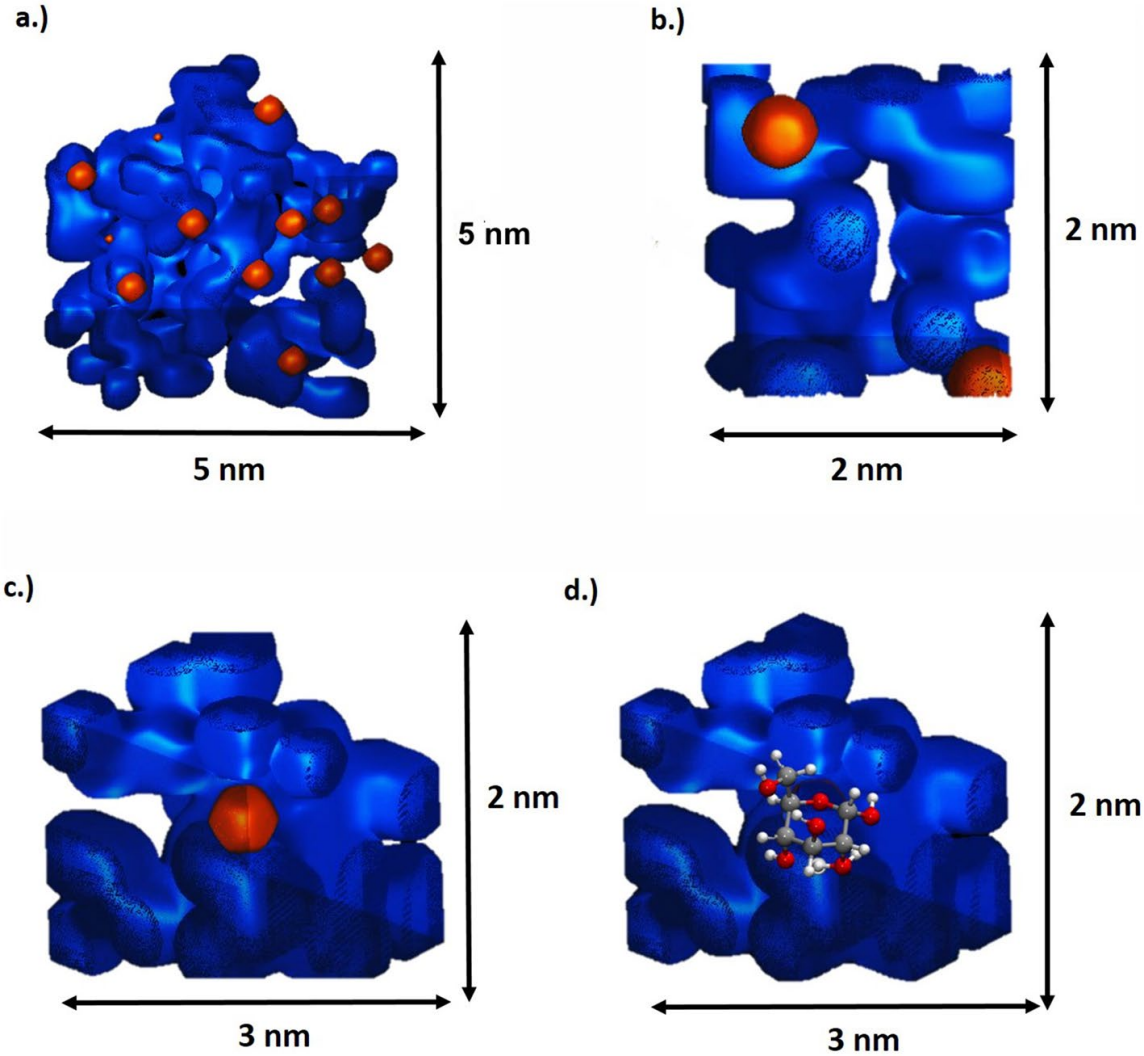
(**a**–**c**) Isosurfaces of water in blue 67% and the complete glucose molecule at m/z = 181 u in orange are shown to visualize the hydrate shell of the solvent glucose molecules. (**d**) DFT calculated molecule structure inserted at the determined glucose molecule position.

In a saturated glucose solution, there are 21 water molecules available to solvate each glucose molecule. In order to visualize the hydrate shell around a solvated glucose molecule, two isosurfaces in a small volume of 5 × 5 nm, 2 × 3 nm, and 2 × 2 nm are plotted (Fig. 10a–c). The position of evaporated glucose molecules is indicated by a second iso-surface. The surrounding water molecules/clusters are represented by the blue iso-surface with a water concentration of around 67% (Fig. 10a–c). The glucose molecules are surrounded by water molecules and homogenously distributed inside the volume. The structural information of the individual molecules is of course not directly retrievable from the APT data. In Fig. 10d, the DFT calculated structure is placed at the position of a detected glucose molecule implying a higher resolution than technically achieved.

More advanced reconstruction algorithms combined with suitable simulation packages and DFT calculations might be able to derive the likely structure on an atomic scale from the purely molecular data sets, if, of course, other problematic sources of error are excluded. Any alteration of the sample structure by the freezing process has to be excluded. This is a necessary precondition like for all characterisation methods. Even though the change of the initial state due to the preparation process cannot be completely excluded, Fig. 10a–c exemplarily shows that there is a minimum distance between the individual glucose molecules. The average minimum distance to the next glucose molecule is 0.75 nm, which can be explained by a surrounding hydration shell around the glucose molecules. This circumstance and the homogeneous overall distribution of the molecules in the total volume indicate that the reconstruction reasonably represents the expected distribution in the solution in a first approximation.

## Summary

Equipped with the knowledge for pure water, we now have provided field evaporation measurements for a saturated glucose solution. Due to the low evaporation field of water, the glucose molecules evaporated as large, partially intact, molecules. Smaller fragments overlap with the signals related to water. However, overlap and ambiguity of mass peaks leaves room for various approaches of chemical interpretation. The APT data of pure bulk glucose differ strongly from the data of the glucose solution. The solid bulk material exhibits a higher fragmentation rate of the molecule rings as compared to the solution, probably caused by a high evaporation field of bulk glucose. Other causes may be different absorption coefficients and/or different binding conditions between bulk glucose and the solution. Nevertheless, with comparison of both measurements and the a-priori knowledge of the overall composition, a suitable interpretation of the mask peaks could be suggested.

A reasonable matching of the measured stoichiometry with the known one can be achieved by interpreting the overlap in the range m/z = 27–36 u and m/z = 41–48 u as a ratio of 33% of C_*x*_O_*y*_H_*z*_ to 67% (H_2_O)_1–5_H^+^, resulting in a total mass mixing ratio of about 0.48, which agrees reasonably with the theoretical value of 0.47. However, there is also a certain overlap with many other peaks, which makes it exceedingly difficult to calculate them exactly, since the exact fractions are not yet known. A significant loss of oxygen or hydrogen cannot be excluded. The identification of glucose molecules within the matrix is only possible by the existence of nearly complete ionic molecules. These allow an unambiguous identification, locally as well, in the volume space. The effective detection efficiency of the solute molecules results in only one molecule out of three identified with certainty.

What sounds like a disadvantage in comparison to the analysis of metals and semiconductors, enables APT to retrieve distinguishable information’s of solutes in aqueous solutions, if a sample modification by the freezing process can be excluded. A breakdown of the solutes into smaller molecular parts would render them invisible against the background of the matrix signals.

A better understanding of the measurement conditions is necessary with regard to the solutes, laser power, field strength, and temperature. The change of molecular fragmentation depending on the local environment has to be understood. Furthermore, the influence of the evaporation of large clusters on the accuracy of the reconstruction is not known and must be examined, and in the best case, be attributed by an improved reconstruction algorithm. Nevertheless, the possibility to detect certain molecules within an aqueous solution, opens the opportunity to inject suitable markers and biological molecules, to study their distribution in various reactions in 3D with sub-nanometre resolution.

## Materials and methods

### Materials

As sample material, glucose (EP, BP, JP, USP testing specifications, anhydrous, from Sigma Aldrich) was used. For the supersaturated glucose solution, water which was ionized and filtered through a Milli-Q system (Millipore) was used. By careful cleaning of all materials and the usage of pure constituents, overlapping in the ToF-mass spectrum from impurities is avoided, since this would lead to an even more challenging peak analysis. To create a saturated glucose solution, glucose was added to water until the solubility limit was reached and a sediment formed on the bottom of the test glass. Tungsten was chosen as a substrate material. To create a rough and reproducible surface we followed the approach described in^15^. For the Lift-Out process, tungsten wires were first etched in 2 mol NaOH solution by applying an AC voltage. Afterwards, the tip was cut down to a flat post using the Focused Ion Beam (FIB).

### Freezing process

As reported in previous work^15^, a solution droplet was dipped with a micro pipette on a precooled tungsten post, which is located within a liquid nitrogen bath to create droplets on top (60–250 µm). Subsequently, the sample holder is transferred as fast as possible into the cooled body of the modified transfer shuttle VCT500 from Leica (T =  − 184 °C) and pumped to a pressure of 6 × 10^−1^ mbar. Shortly thereafter, the shuttle is attached to the high vacuum-coater (Leica EM ACE600), to carry out a freeze-etching process to remove ice crystals that were formed by the contact of the sample with air. By heating up the sample very precisely to a temperature of – 90 °C and a pressure of 9 × 10^−7^ mbar for 30 min, a sublimation process from solid ice to vapor occurs, which is necessary for the controlled removal of condensed ice from the sample. In addition, the Leica EM ACE600 high vacuum coater is useful to improve the vacuum condition inside the shuttle (10^−4^ mbar), which is necessary to enable the transfer of the sample into the Focused-Ion-Beam (FIB) Microscope.

### Cryo FIB preparation

In order to prepare cryogenic samples in the FIB (FEI Scios) into nano-shaped tips with an apex radius less than 100 nm, which is required for the APT method, the FIB has to be equipped with a custom made cryo-stage, which is cooled down to a temperature of − 150 °C using copper bands connected to a N_2_ Dewar attached to the microscope. The sample itself is transferred into the microscope using a dedicated VCT500 load lock. This load lock was mounted at the back side of the SEM to the port intentional designed for the STEM detector, which allows an easy sample transport into the cryo-stage. Cold surfaces act as a trap for surrounding gas or molecules. To avoid re-deposition of material onto the shaped sample, a cryo-shield was additionally installed in the chamber.

### Cryo APT specimen preparation

**Figure 11:**
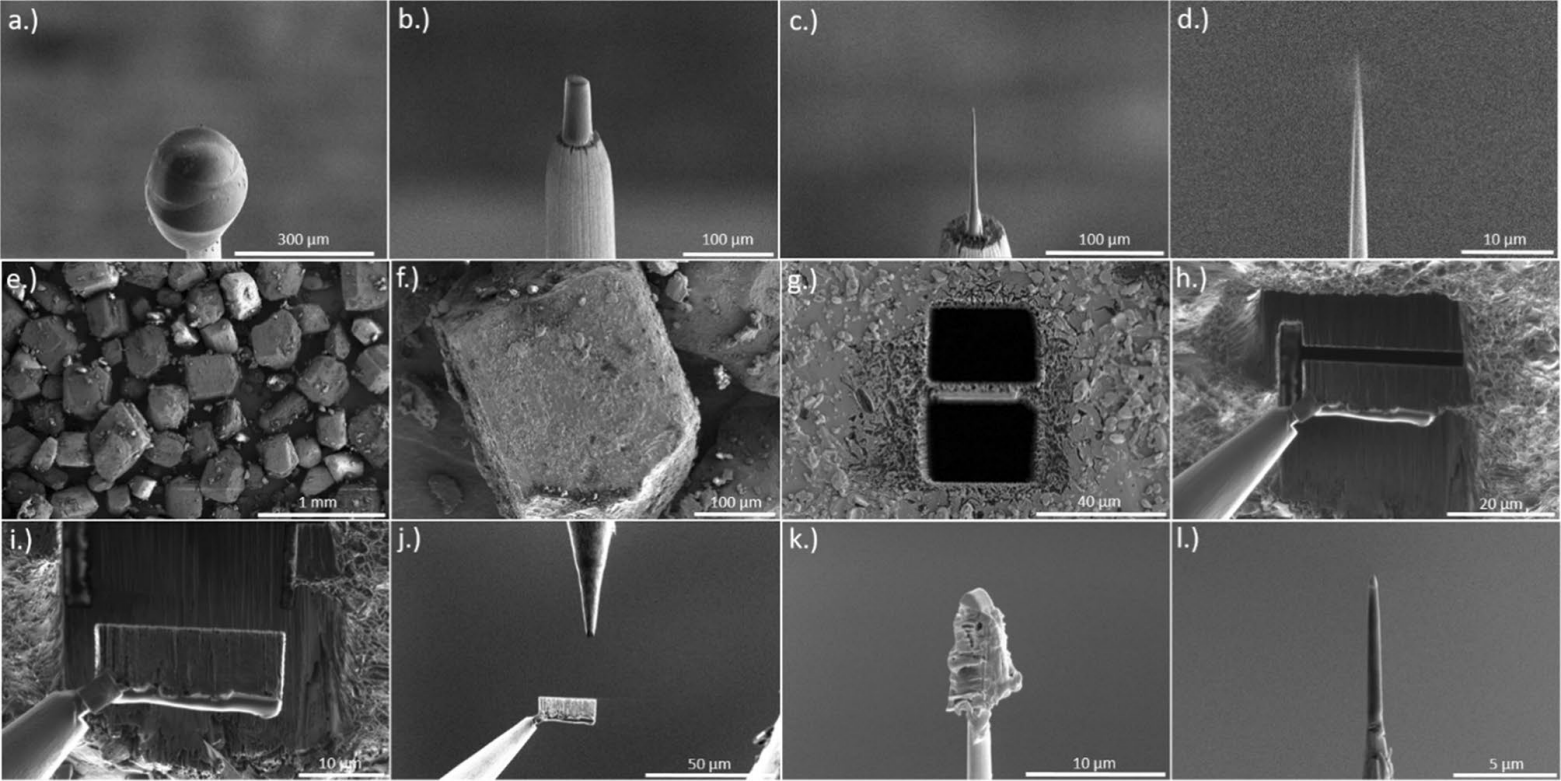
FIB preparation—(**a**) Frozen droplet of a saturated glucose solution on top of a tungsten post. (**b**–**d**) Milling process with an annular pattern with decreasing inner diameter until a tip radius < 100 nm (**d**) occurs. From (**e**) to (**l**) the lift-out procedure from a glucose bulk sample is shown, followed by an annular milling process to the final obtained tip (**l**).

SEM imaging was typically performed with low energy (5 kV 25 pA) to prevent melting of the sample by electron bombardment. For the milling process, the sample has been tilted to 52°, which aligns it vertically towards the ion beam. A circular ring pattern was used for azimuthal milling. Initial milling steps were performed at 30 kV acceleration voltage and an ion beam current of 50 nA, until the tungsten substrate became visible again. After reaching a radius of 30 µm, the beam current is gradually reduced with decreasing radius, down to an inner ring diameter of 300 nm and a beam current of 0.1 pA. The thinning process is monitored by taking snapshots using the electron beam. The shaping process continues until a very sharp tip with a radius < 100 nm (Fig. 11a–d) is obtained. The finished tip is then transferred back into the shuttle and can be attached to the APT. To our experience reproducible measurements with sufficient throughput require a high aspect ratio of the tip. A tip length of several tens of µm is desirable. The preparation path for a cryo-APT specimen is depicted in Fig. 11a–d.

### APT specimen preparation

The glucose crystals were glued with a silver glue (RS pro silver conductive glue) on a SEM grid. For better conductivity, and to minimalize the charging effect, 50 nm copper was sputtered on top with the high vacuum-coater (Leica EM ACE600) by glow discharge sputtering. Deposition parameter were 60 mA current and an Ar pressure of 2.0 × 10^−2^ mbar and an effective sputter rate of 0.15 nm/s. Afterwards, a normal APT specimen Lift-Out method was used^46^ (see Fig. 11e–l), followed by a shaping process.

### APT measurements

Using a custom-made atom probe^56^ equipped with NOPA to continuously change wavelength in the range between 350 and 900 nm, measurements were conducted with a laser wavelength of 355 nm. The pulse length was chosen to 250 fs. The spot size in the focus point amounts to a diameter of 50 microns. As detector, a 120 mm diameter delay line detector with chevron MCP setup with an open area ratio (OAR) of 50% is used. The flight length amounts to 130 mm and the effective half angle of the field of view to 38°. The system is equipped with a custom-made cryo-transfer port to accept a standard VCT500 from Leica for the transfer of cryogenic samples. The exchange of the sample in the buffer chamber uses a PEEK isolated storage position to avoid melting of the sample during transfer. The obtained datasets were analysed and reconstructed using the Scito^57^ software package. Calculated mass spectra were exported into csv files and plotted using OriginPro^58^ for publication.

### Reconstruction

The volume was reconstructed using a geometrical reconstruction algorithm^59^ based on the original point projection method by Bas et al.^60^. SEM pictures were used to determine the initial radius of the tip. This radius and the taper angle were used to optimize the calculated evaporation-field curve. All necessary parameters, such as field and image compression factors were determined in earlier experiments. For the z-axis reconstruction, the respective densities of each molecular fragment were calculated to achieve the correct density values after splitting of the assumed molecule in its atomic constituents.

### DFT calculation

To explain the obtained fragments, we performed calculations with density functional theory (DFT) to study the stability of several ions that can explain the observed m/z ratios. All energies reported were obtained with the functional BP86^61,62^ and the basis set def2-SVP^63,64^, with D3 dispersion correction^65^. The calculations were performed with Turbomole^66,67^ run through ChemShell^68,69^. Harmonic frequency calculations confirmed the structures as minima on the potential energy surface. The energies include the harmonic zero-point vibrational energy. All calculations were performed in the absence of an external electric field. Partial charges were analysed using natural bond orbitals (NBO)^70^.

The starting geometry for the calculation was an α-D-glucopyranose molecule. The geometry at m/z = 181 u is equivalent to a glucose molecule with an additional proton. The proton was attached to different atoms in the molecule in order to find the most likely binding site. The geometry with the lowest energy found the additional H to be bound between two oxygen atoms.

By adding a hydronium cation to glucose, the geometry for m/z = 199 u is obtained. The hydronium can approach the glucose molecule from several different directions, thereby resulting in a multitude of possible molecular structures. The structure with the lowest energy is chosen as the most likely and serves as the starting geometry for the calculation of the next structure at m/z = 217 u, in which case H_2_O is added. The structure of glucose + (H_2_O)_16_H^+^ is the result of an MD simulation with GFN-xTB^71^, followed by an energy minimization with BP86/def2-SVP.

The simulation of structures at mass-to-charge state ratios of m/z = 163 u and below progressed in a similar fashion. One hydrogen and an OH group were removed from the glucose molecule. Several different possible combinations were investigated. Each structure with the lowest energy served as starting point for the structure with the next lower mass-to-charge state ratio.

## Supplementary information

Supplementary Information.
