# Supplementary material for: 3D sub-nanometer analysis of glucose in an aqueous solution by cryo-atom probe tomography
Source: Sci Rep. 2021 Jun 2;11:11607. doi: 10.1038/s41598-021-90862-8 (PMC8172843; doi:10.1038/s41598-021-90862-8)
Supplement: Supplementary file 1 — Supplementary Information. [file 41598_2021_90862_MOESM1_ESM.docx]

**Supplementary Part**

**3D Sub-Nanometer Analysis of Glucose in an Aqueous Solution by Cryo-Atom Probe Tomography**

T. M. Schwarz^1^, C. A. Dietrich^2,^ J. Ott^1^, E. M. Weikum^1^, R. Lawitzki^1^, I. Balla^1^, E. Hadjixenophontos^1^, J. Kästner^2^, G. Schmitz^1^, P. Stender^1*^,

1. University of Stuttgart, Institute for Materials Science, Chair of Materials Physics, Heisenbergstr. 3, 70569 Stuttgart, Germany
2. University of Stuttgart, Institute for Theoretical Chemistry, Pfaffenwaldring 55, 70569 Stuttgart, Germany

*Corresponding author E-mail address: [patrick.stender@imw.uni-stuttgart.de](mailto:patrick.stender@imw.uni-stuttgart.de)

Table A 1: Possible combinations of different C_x_H_y_, C_x_O_y_H_z_ and H_x_O_y_ molecules in a supersaturated glucose solution considering only the single charged state. If double-charged molecules were taken into account, the combination possiblities would increase enormously.

| m/q | molecules |  |  |  |  | m/q | molecules |  |  |  |
| --- | --- | --- | --- | --- | --- | --- | --- | --- | --- | --- |
| 1 | H |  |  |  |  | **96** | C6OH8 | C5O2H4 | C4O3 |  |
| 2 | H2 |  |  |  |  | **97** | C6OH9 | C5O2H5 | C4O3H |  |
| 3 | H3 |  |  |  |  | **98** | C6OH10 | C5O2H6 | C4O3H2 |  |
| 12 | C |  |  |  |  | **99** | C6OH11 | C5O2H7 | C4O3H3 |  |
| 15 | CH3 |  |  |  |  | **100** | C6OH12 | C5O2H8 | C4O3H4 |  |
| 16 | O |  |  |  |  | **102** |  | C5O2H10 | C4O3H6 |  |
| 17 | HO |  |  |  |  | **103** |  | C5O2H11 | C4O3H7 |  |
| 18 | H2O |  |  |  |  | **104** | C6O2 | C5O2H12 | C4O3H8 |  |
| 19 | H3O |  |  |  |  | **105** | C6O2H | C5O2H13 | C4O3H9 |  |
| 20 | H4O |  |  |  |  | **106** | C6O2H2 |  | C4O3H10 |  |
| 21 | H5O |  |  |  |  | **107** | C6O2H3 |  | C4O3H11 |  |
| 27 | C2H3 |  |  |  |  | **108** | C6O2H4 |  | C5O3 |  |
| 28 | C2H4 | CO |  |  |  | **109** | C6O2H5 |  | C5O3H |  |
| 29 | C2H5 | COH |  |  |  | **110** | C6O2H6 |  | C5O3H2 |  |
| 30 | C2H6 | COH2 |  |  |  | **111** | C6O2H7 |  | C5O3H3 |  |
| 31 | C2H7 | COH3 |  |  |  | **112** | C6O2H8 |  | C5O3H4 |  |
| 32 | O2 | COH4 |  |  |  | **113** | C6O2H9 | C4O4H | C5O3H5 |  |
| 33 | O2H |  |  |  |  | **114** | C6O2H10 | C4O4H2 | C5O3H6 |  |
| 34 | (OH)2 |  |  |  |  | **115** | C6O2H11 | C4O4H3 | C5O3H7 |  |
| 35 | (OH)(OH2) |  |  |  |  | **116** | C6O2H12 | C4O4H4 | C5O3H8 |  |
| 36 | C3 |  |  |  |  | **117** | C6O2H13 | C4O4H5 | C5O3H9 |  |
| 37 | (H2O)(H3O) | C3H |  |  |  | **118** |  | C4O4H6 | C5O3H10 |  |
| 39 | C3H3 |  |  |  |  | **119** |  | C4O4H7 | C5O3H11 |  |
| 40 | C3H4 | C2O |  |  |  | **120** | C6O3 | C4O4H8 | C5O3H12 |  |
| 41 | C3H5 | C2OH |  |  |  | **121** | C6O3H | C4O4H9 | C5O3H13 |  |
| 42 | C3H6 | C2OH2 |  |  |  | **122** | C6O3H2 | C4O4H10 |  |  |
| 43 | C3H7 | C2OH3 |  |  |  | **123** | C6O3H3 |  |  |  |
| 44 | C3H8 | C2OH4 | CO2 |  |  | **124** | C6O3H4 | C5O4 |  |  |
| 45 | C3H9 | C2OH5 | CO2H |  |  | **125** | C6O3H5 | C5O4H |  |  |
| 46 |  | C2OH6 | CO2H2 |  |  | **126** | C6O3H6 | C5O4H2 |  |  |
| 47 |  | C2OH7 | CO2H3 |  |  | **127** | C6O3H7 | C5O4H3 |  |  |
| 48 | C4 |  | CO2H4 |  |  | **128** | C6O3H8 | C5O4H4 |  |  |
| 49 | C4H |  | CO2H5 |  |  | **129** | C6O3H9 | C5O4H5 |  |  |
| 52 | C4H4 |  | C3O |  |  | **130** | C6O3H10 | C5O4H6 |  |  |
| 54 | C4H6 |  | C3OH2 |  |  | **131** | C6O3H11 | C5O4H7 |  |  |
| 55 | (H2O)2(H3O) | C4H7 | C3OH3 |  |  | **132** | C6O3H12 | C5O4H8 |  |  |
| 56 | C4H8 |  | C3OH4 | C2O2 |  | **133** | C6O3H13 | C5O4H9 |  |  |
| 57 | C4H9 |  | C3OH5 | C2O2H |  | **134** |  | C5O4H10 |  |  |
| 58 | C4H10 |  | C3OH6 | C2O2H2 |  | **135** |  | C5O4H11 |  |  |
| 59 | C4H11 |  | C3OH7 | C2O2H3 |  | **136** | C6O4 | C5O4H12 |  |  |
| 60 | C5 |  | C3OH8 | C2O2H4 |  | **137** | C6O4H | C5O4H13 |  |  |
| 61 | C5H |  | C3OH9 | C2O2H5 |  | **138** | C6O4H2 | C5O4H14 |  |  |
| 62 | C5H2 |  |  | C2O2H6 |  | **139** | C6O4H3 |  |  |  |
| 63 | C5H3 |  |  | C2O2H7 |  | **140** | C6O4H4 | C5O5 |  |  |
| 64 | C5H4 |  |  | C4O |  | **141** | C6O4H5 | C5O5H |  |  |
| 65 | C5H5 |  |  | C4OH |  | **142** | C6O4H6 | C5O5H2 |  |  |
| 66 | C5H6 |  |  | C4OH2 |  | **143** | C6O4H7 | C5O5H3 |  |  |
| 67 | C5H7 |  |  | C4OH3 |  | **144** | C6O4H8 | C5O5H4 |  |  |
| 68 | C5H8 |  |  | C4OH4 | C3O2 | **145** | C6O4H9 | C5O5H5 |  |  |
| 69 | C5H9 |  |  | C4OH5 | C3O2H | **147** | C6O4H11 | C5O5H7 |  |  |
| 70 | C5H10 |  |  | C4OH6 | C3O2H2 | **148** | C6O4H12 | C5O5H8 |  |  |
| 71 | C5H11 |  |  | C4OH7 | C3O2H3 | **149** | C6O4H13 | C5O5H9 |  |  |
| 72 | C5H12 | C6 |  | C4OH8 | C3O2H4 | **150** | C6O4H14 | C5O5H10 |  |  |
| 73 | (H2O)3(H30) | C6H | C5H13 | C4OH9 | C3O2H5 | **151** |  | C5O5H11 |  |  |
| 77 | C5OH | C6H5 |  |  | C3O2H9 | **152** | C6O5 | C5O5H12 |  |  |
| 78 | C5OH2 | C6H6 |  |  |  | **153** | C6O5H | C5O5H13 |  |  |
| 79 | C5OH3 | C6H7 |  |  |  | **157** | C6O5H5 |  |  |  |
| 80 | C5OH4 | C6H8 | C4O2 |  |  | **163** | C6O5H11 |  |  |  |
| 81 | C5OH5 | C6H9 | C4O2H |  |  | **169** | C6O6H |  |  |  |
| 82 | C5OH6 | C6H10 | C4O2H2 |  |  | **171** | C6O6H3 |  |  |  |
| 83 | C5OH7 | C6H11 | C4O2H3 |  |  | **175** | C6O6H7 |  |  |  |
| 84 | C5OH8 | C6H12 | C4O2H4 |  |  | **181** | C6O6H13 |  |  |  |
| 85 | C5OH9 | C6H13 | C4O2H5 |  |  | **187** | C5O5H10+H2O+H3O | |  |  |
| 86 | C5OH10 | C6H14 | C4O2H6 |  |  | **189** | C5O5H12+H2O+H3O | |  |  |
| 87 | C5OH11 | C6H15 | C4O2H7 |  |  | **193** | C5O5H15+H3O+H3O | |  |  |
| 91 | (H20)4(H3O) | C6OH3 | C4O2H11 |  |  | **199** | C6O6H12+H3O |  |  |  |
| 92 |  | C6OH4 |  | C5O2 |  | **205** | C5O5H11+H2O+H2O+H2O | |  |  |
| 93 | C6OH5 | C5O2H |  |  |  | **207** | C5O5H13+H2O+H2O+H2O | |  |  |
| 94 | C6OH6 | C5O2H2 |  |  |  | **211** | C5O5H14+H3O+H3O+H3O | |  |  |
| 95 | C6OH7 | C5O2H3 |  |  |  | **217** | C6O6H12+H2O+H3O | |  |  |

Table A 2: Possible combinations of different C_x_H_y_, C_x_O_y_H_z_ molecules for a bulk glucose specimen considering only the single charged state.

|  |  | |  |  | Approach 1 | Approach 2 |
| --- | --- | --- | --- | --- | --- | --- |
| m/q | **molecules** | |  |  | **C*_x_*H*_y_*** | **C*_x_*O*_y_*H*_z_*** |
| 1 | H |  |  |  | H | H |
| 2 | H2 |  |  |  | H2 | H2 |
| 3 | H3 |  |  |  | H3 | H3 |
| 12 | C |  |  |  | C | C |
| 13 | CH |  |  |  | CH | CH |
| 14 | CH2 |  |  |  | CH2 | CH2 |
| 15 | CH3 |  |  |  | CH3 | CH3 |
| 16 | O |  |  |  | O | O |
| 17 | OH |  |  |  | OH | OH |
| 18 | OH2 |  |  |  | OH2 | OH2 |
| 19 | OH3 |  |  |  | OH3 | OH3 |
| 20 | OH4 |  |  |  | OH4 | OH4 |
| 21 | OH5 |  |  |  | OH5 | OH5 |
| 26 | C2H2 |  |  |  | C2H2 | C2H2 |
| 27 | C2H3 |  |  |  | C2H3 | C2H3 |
| 28 | C2H4 | CO |  |  | C2H4 | CO |
| 29 | C2H5 | COH |  |  | C2H5 | COH |
| 30 | C2H6 | COH2 |  |  | C2H6 | COH2 |
| 31 | C2H7 | COH3 |  |  | C2H7 | COH3 |
| 32 | O2 | COH4 |  |  | O2 | O2 |
| 36 | C3 |  |  |  | C3 | C3 |
| 37 | C3H |  |  |  | C3H | C3H |
| 38 | C3H2 |  |  |  | C3H2 | C3H2 |
| 39 | C3H3 |  |  |  | C3H3 | C3H3 |
| 40 | C3H4 | C2O |  |  | C3H4 | C2O |
| 41 | C3H5 | C2OH |  |  | C3H5 | C2OH |
| 42 | C3H6 | C2OH2 |  |  | C3H6 | C2OH2 |
| 43 | C3H7 | C2OH3 |  |  | C3H7 | C2OH3 |
| 44 | C3H8 | C2OH4 | CO2 |  | C3H8 | CO2 |
| 45 |  | C2OH5 | CO2H |  | C2OH5 | CO2H |
| 46 |  | C2OH6 | CO2H2 |  | C2OH6 | CO2H2 |
| 47 |  | C2OH7 | CO2H3 |  | C2OH7 | CO2H3 |
| 48 | C4 |  | CO2H4 |  | C4 | CO2H4 |
| 49 | C4H |  | CO2H5 |  | C4H | CO2H5 |
| 50 | C4H2 |  |  |  | C4H2 | C4H2 |
| 51 | C4H3 |  |  |  | C4H3 | C4H3 |
| 52 | C4H4 | C3O |  |  | C4H4 | C3O |
| 53 | C4H5 | C3OH |  |  | C4H5 | C3OH |
| 54 | C4H6 | C3OH2 |  |  | C4H6 | C3OH2 |
| 55 | C4H7 | C3OH3 |  |  | C4H7 | C3OH3 |
| 56 | C4H8 | C3OH4 | C2O2 |  | C4H8 | C2O2 |
| 57 | C4H9 | C3OH5 | C2O2H |  | C4H9 | C2O2H |
| 58 | C4H10 | C3OH6 | C2O2H2 |  | C4H10 | C2O2H2 |
| 59 |  | C3OH7 | C2O2H3 |  | C3OH7 | C2O2H3 |
| 60 | C5 | C3OH8 | C2O2H4 |  | C5 | C2O2H4 |
| 61 | C5H | C3OH9 | C2O2H5 |  | C5H | C2O2H5 |
| 62 | C5H2 |  | C2O2H6 |  | C5H2 | C2O2H6 |
| 63 | C5H3 |  | C2O2H7 |  | C5H3 | C2O2H7 |
| 64 | C5H4 |  | C4O |  | C5H4 | C4O |
| 65 | C5H5 |  | C4OH |  | C5H5 | C4OH |
| 66 | C5H6 |  | C4OH2 |  | C5H6 | C4OH2 |
| 67 | C5H7 |  | C4OH3 |  | C5H7 | C4OH3 |
| 68 | C5H8 |  | C4OH4 | C3O2 | C5H8 | C3O2 |
| 69 | C5H9 |  | C4OH5 | C3O2H | C5H9 | C3O2H |
| 70 | C5H10 |  | C4OH6 | C3O2H2 | C5H10 | C3O2H2 |
| 71 | C5H11 |  | C4OH7 | C3O2H3 | C5H11 | C3O2H3 |
| 72 | C5H12 | C6 | C4OH8 | C3O2H4 | C6 | C3O2H4 |
| 73 |  | C6H | C4OH9 | C3O2H5 | C6H | C3O2H5 |
| 74 |  | C6H2 |  | C3O2H9 | C6H2 | C3O2H9 |
| 75 |  | C6H3 |  |  | C6H3 | C6H3 |

Table A 3: For the frist approach only C_x_H_y_ molecules are considered. In the second approach C_x_H_y_ molecules and common protonated water cluster with the formula (H_2_O)_n_H^+^ with n = 1 -5 were assumed.

| Approach 1 | |  |  |  |  | Approach 2 | |  |  |
| --- | --- | --- | --- | --- | --- | --- | --- | --- | --- |
| m/q | **molecules** | **m/q** | **molecules** | |  | **m/q** | **molecules** | **m/q** | **molecules** |
| 1 | H | **96** | C6OH8 |  |  | **1** | H | **96** | C6OH8 |
| 2 | H2 | **97** | C6OH9 |  |  | **2** | H2 | **97** | C6OH9 |
| 3 | H3 | **98** | C6OH10 |  |  | **3** | H3 | **98** | C6OH10 |
| 12 | C | **99** | C6OH11 |  |  | **12** | C | **99** | C6OH11 |
| 15 | CH3 | **100** | C6OH12 |  |  | **15** | CH3 | **100** | C6OH12 |
| 16 | O | **102** | C6OH14 |  |  | **16** | O | **102** | C6OH14 |
| 17 | HO | **103** | C5O2H11 |  |  | **17** | HO | **103** | C5O2H11 |
| 18 | H2O | **104** | C6O2 |  |  | **18** | H2O | **104** | C6O2 |
| 19 | H3O | **105** | C6O2H |  |  | **19** | H3O | **105** | C6O2H |
| 20 | H4O | **106** | C6O2H2 |  |  | **20** | H4O | **106** | C6O2H2 |
| 21 | H5O | **107** | C6O2H3 |  |  | **21** | H5O | **107** | C6O2H3 |
| 27 | C2H3 | **108** | C6O2H4 |  |  | **27** | C2H3 | **108** | C6O2H4 |
| 28 | C2H4 | **109** | C6O2H5 |  |  | **28** | C2H4 | **109** | C6O2H5 |
| 29 | C2H5 | **110** | C6O2H6 |  |  | **29** | C2H5 | **110** | C6O2H6 |
| 30 | C2H6 | **111** | C6O2H7 |  |  | **30** | C2H6 | **111** | C6O2H7 |
| 31 | C2H7 | **112** | C6O2H8 |  |  | **31** | C2H7 | **112** | C6O2H8 |
| 32 | O2 | **113** | C6O2H9 |  |  | **32** | O2 | **113** | C6O2H9 |
| 33 | O2H | **114** | C6O2H10 |  |  | **33** | O2H | **114** | C6O2H10 |
| 34 | (OH)2 | **115** | C6O2H11 |  |  | **34** | (OH)2 | **115** | C6O2H11 |
| 35 | (OH)(OH2) | **116** | C6O2H12 |  |  | **35** | (OH)(OH2) | **116** | C6O2H12 |
| 36 | C3 | **117** | C6O2H13 |  |  | **36** | C3 | **117** | C6O2H13 |
| 37 | C3H | **118** | C6O2H14 |  |  | **37** | (H20)(H3O) | **118** | C6O2H14 |
| 39 | C3H3 | **119** | C5O3H11 |  |  | **39** | C3H3 | **119** | C5O3H11 |
| 40 | C3H4 | **120** | C6O3 |  |  | **40** | C3H4 | **120** | C6O3 |
| 41 | C3H5 | **121** | C6O3H |  |  | **41** | C3H5 | **121** | C6O3H |
| 42 | C3H6 | **122** | C6O3H2 |  |  | **42** | C3H6 | **122** | C6O3H2 |
| 43 | C3H7 | **123** | C6O3H3 |  |  | **43** | C3H7 | **123** | C6O3H3 |
| 44 | C3H8 | **124** | C6O3H4 |  |  | **44** | C3H8 | **124** | C6O3H4 |
| 45 | C3H9 | **125** | C6O3H5 |  |  | **45** | C3H9 | **125** | C6O3H5 |
| 46 | C2OH6 | **126** | C6O3H6 |  |  | **46** | C2OH6 | **126** | C6O3H6 |
| 47 | C2OH7 | **127** | C6O3H7 |  |  | **47** | C2OH7 | **127** | C6O3H7 |
| 48 | C4 | **128** | C6O3H8 |  |  | **48** | C4 | **128** | C6O3H8 |
| 49 | C4H | **129** | C6O3H9 |  |  | **49** | C4H | **129** | C6O3H9 |
| 52 | C4H4 | **130** | C6O3H10 |  |  | **52** | C4H4 | **130** | C6O3H10 |
| 54 | C4H6 | **131** | C6O3H11 |  |  | **54** | C4H6 | **131** | C6O3H11 |
| 55 | C4H7 | **132** | C6O3H12 |  |  | **55** | (H20)2(H3O) | **132** | C6O3H12 |
| 56 | C4H8 | **133** | C6O3H13 |  |  | **56** | C4H8 | **133** | C6O3H13 |
| 57 | C4H9 | **134** | C5O4H10 |  |  | **57** | C4H9 | **134** | C5O4H10 |
| 58 | C4H10 | **135** | C5O4H11 |  |  | **58** | C4H10 | **135** | C5O4H11 |
| 59 | C4H11 | **136** | C6O4 |  |  | **59** | C4H11 | **136** | C6O4 |
| 60 | C5 | **137** | C6O4H |  |  | **60** | C5 | **137** | C6O4H |
| 61 | C5H | **138** | C6O4H2 |  |  | **61** | C5H | **138** | C6O4H2 |
| 62 | C5H2 | **139** | C6O4H3 |  |  | **62** | C5H2 | **139** | C6O4H3 |
| 63 | C5H3 | **140** | C6O4H4 |  |  | **63** | C5H3 | **140** | C6O4H4 |
| 64 | C5H4 | **141** | C6O4H5 |  |  | **64** | C5H4 | **141** | C6O4H5 |
| 65 | C5H5 | **142** | C6O4H6 |  |  | **65** | C5H5 | **142** | C6O4H6 |
| 66 | C5H6 | **143** | C6O4H7 |  |  | **66** | C5H6 | **143** | C6O4H7 |
| 67 | C5H7 | **144** | C6O4H8 |  |  | **67** | C5H7 | **144** | C6O4H8 |
| 68 | C5H8 | **145** | C6O4H9 |  |  | **68** | C5H8 | **145** | C6O4H9 |
| 69 | C5H9 | **147** | C6O4H11 |  |  | **69** | C5H9 | **147** | C6O4H11 |
| 70 | C5H10 | **148** | C6O4H12 |  |  | **70** | C5H10 | **148** | C6O4H12 |
| 71 | C5H11 | **149** | C6O4H13 |  |  | **71** | C5H11 | **149** | C6O4H13 |
| 72 | C5H12 | **150** | C6O4H14 |  |  | **72** | C5H12 | **150** | C6O4H14 |
| 73 | C5H13 | **151** | C5O5H11 |  |  | **73** | (H2O)3(H30) | **151** | C5O5H11 |
| 77 | C6H5 | **152** | C6O5 |  |  | **77** | C6H5 | **152** | C6O5 |
| 78 | C6H6 | **153** | C6O5H |  |  | **78** | C6H6 | **153** | C6O5H |
| 79 | C6H7 | **157** | C6O5H5 |  |  | **79** | C6H7 | **157** | C6O5H5 |
| 80 | C6H8 | **163** | C6O5H11 |  |  | **80** | C6H8 | **163** | C6O5H11 |
| 81 | C6H9 | **169** | C6O6H |  |  | **81** | C6H9 | **169** | C6O6H |
| 82 | C6H10 | **171** | C6O6H3 |  |  | **82** | C6H10 | **171** | C6O6H3 |
| 83 | C6H11 | **175** | C6O6H7 |  |  | **83** | C6H11 | **175** | C6O6H7 |
| 84 | C6H12 | **181** | C6O6H13 |  |  | **84** | C6H12 | **181** | C6O6H13 |
| 85 | C6H13 | **187** | C5O5H10+H2O+H3O | |  | **85** | C6H13 | **187** | C5O5H10+H2O+H3O |
| 86 | C6H14 | **189** | C5O5H12+H2O+H3O | |  | **86** | C6H14 | **189** | C5O5H12+H2O+H3O |
| 87 | C6H15 | **193** | C5O5H15+H3O+H3O | |  | **87** | C6H15 | **193** | C5O5H15+H3O+H3O |
| 91 | C6OH3 | **199** | C6O6H12+H3O | |  | **91** | (H2O)4(H30) | **199** | C6O6H12+H3O |
| 92 | C6OH4 | **205** | C5O5H11+H2O+H2O+H2O | | **92** | | C6OH4 | **205** | C5O5H11+H2O+H2O+H2O |
| 93 | C6OH5 | **207** | C5O5H13+H2O+H2O+H2O | | **93** | | C6OH5 | **207** | C5O5H13+H2O+H2O+H2O |
| 94 | C6OH6 | **211** | C5O5H14+H3O+H3O+H3O | | **94** | | C6OH6 | **211** | C5O5H14+H3O+H3O+H3O |
| 95 | C6OH7 | **217** | C6O6H12+H2O+H3O | |  | **95** | C6OH7 | **217** | C6O6H12+H2O+H3O |

Table A 4: In the third approach C_x_O_y_H_z_ molecules and common protonated water cluster with the formula (H_2_O)_n_H^+^ with n = 1 -5 were assumed. In the fourth appraoch, the same molecules were assumed as in the third approach, in addition, the overlap with water peaks in the mass-to-carge state ratio of 27-31, 36 and 41-48 u e^-1^ were calculated. The overlaping regions are marked in red.

| Appraoch 3 | |  |  |  | | Approach 4 | | |  | |  |  | |  | |
| --- | --- | --- | --- | --- | --- | --- | --- | --- | --- | --- | --- | --- | --- | --- | --- |
| m/q | **molecules** | **m/q** | **molecules** | | **m/q** | | **molecules** | | | **m/q** | | **molecules** |  | |  |
| 1 | H | **96** | C6OH8 |  | | **1** | H | |  | | **96** | C6OH8 | |  | |
| 2 | H2 | **97** | C6OH9 |  | | **2** | H2 | |  | | **97** | C6OH9 | |  | |
| 3 | H3 | **98** | C6OH10 |  | | **3** | H3 | |  | | **98** | C6OH10 | |  | |
| 12 | C | **99** | C6OH11 |  | | **12** | C | |  | | **99** | C6OH11 | |  | |
| 15 | CH3 | **100** | C6OH12 |  | | **15** | CH3 | |  | | **100** | C6OH12 | |  | |
| 16 | O | **102** | C4O3H6 |  | | **16** | O | |  | | **102** | C4O3H6 | |  | |
| 17 | HO | **103** | C4O3H7 |  | | **17** | HO | |  | | **103** | C4O3H7 | |  | |
| 18 | H2O | **104** | C6O2 |  | | **18** | H2O | |  | | **104** | C6O2 | |  | |
| 19 | H3O | **105** | C6O2H |  | | **19** | H3O | |  | | **105** | C6O2H | |  | |
| 20 | H4O | **106** | C6O2H2 |  | | **20** | H4O | |  | | **106** | C6O2H2 | |  | |
| 21 | H5O | **107** | C6O2H3 |  | | **21** | H5O | |  | | **107** | C6O2H3 | |  | |
| 27 | C2H3 | **108** | C6O2H4 |  | | **27** | C2H3 | | (OH2)3 | | **108** | C6O2H4 | |  | |
| 28 | CO | **109** | C6O2H5 |  | | **28** | CO | | (OH2)(OH3)2 | | **109** | C6O2H5 | |  | |
| 29 | COH | **110** | C6O2H6 |  | | **29** | COH | | (OH)3(OH2)2 | | **110** | C6O2H6 | |  | |
| 30 | COH2 | **111** | C6O2H7 |  | | **30** | COH2 | | (OH2)5 | | **111** | C6O2H7 | |  | |
| 31 | COH3 | **112** | C6O2H8 |  | | **31** | COH3 | | (OH)(OH3)4 | | **112** | C6O2H8 | |  | |
| 32 | O2 | **113** | C6O2H9 |  | | **32** | O2 | |  | | **113** | C6O2H9 | |  | |
| 33 | O2H | **114** | C6O2H10 |  | | **33** | O2H | |  | | **114** | C6O2H10 | |  | |
| 34 | (OH)2 | **115** | C6O2H11 |  | | **34** | (OH)2 | |  | | **115** | C6O2H11 | |  | |
| 35 | (OH)(OH2) | **116** | C6O2H12 |  | | **35** | (OH)(OH2) | | | | **116** | C6O2H12 | |  | |
| 36 | C3 | **117** | C6O2H13 |  | | **36** | C3 | | (OH2)2 | | **117** | C6O2H13 | |  | |
| 37 | (H20)(H3O) | **118** | C6O2H14 |  | | **37** | (H20)(H3O) | | | | **118** | C6O2H14 | |  | |
| 39 | C3H3 | **119** | C5O3H11 |  | | **39** | C3H3 | |  | | **119** | C5O3H11 | |  | |
| 40 | C2O | **120** | C6O3 |  | | **40** | C2O | |  | | **120** | C6O3 | |  | |
| 41 | C2OH | **121** | C6O3H |  | | **41** | C2OH | | (OH)2O3 | | **121** | C6O3H | |  | |
| 42 | C2OH2 | **122** | C6O3H2 |  | | **42** | C2OH2 | | (OH)4O | | **122** | C6O3H2 | |  | |
| 43 | C2OH3 | **123** | C6O3H3 |  | | **43** | C2OH3 | | (OH)4(OH2) | | **123** | C6O3H3 | |  | |
| 44 | C02 | **124** | C6O3H4 |  | | **44** | C02 | | (OH)2(OH2)3 | | **124** | C6O3H4 | |  | |
| 45 | C02H | **125** | C6O3H5 |  | | **45** | C02H | | (OH2)5 | | **125** | C6O3H5 | |  | |
| 46 | C02H2 | **126** | C6O3H6 |  | | **46** | C02H2 | | (OH2)3(OH3) | | **126** | C6O3H6 | |  | |
| 47 | C02H3 | **127** | C6O3H7 |  | | **47** | C02H3 | | (OH2)(OH3)4 | | **127** | C6O3H7 | |  | |
| 48 | C02H4 | **128** | C6O3H8 |  | | **48** | C02H4 | | (OH3)5H | | **128** | C6O3H8 | |  | |
| 49 | CO2H5 | **129** | C6O3H9 |  | | **49** | CO2H5 | |  | | **129** | C6O3H9 | |  | |
| 52 | C3O | **130** | C6O3H10 |  | | **52** | C3O | |  | | **130** | C6O3H10 | |  | |
| 54 | C3OH2 | **131** | C6O3H11 |  | | **54** | C3OH2 | |  | | **131** | C6O3H11 | |  | |
| 55 | (H2O)2(H30) | **132** | C6O3H12 |  | | **55** | (H2O)2(H30) | | | | **132** | C6O3H12 | |  | |
| 56 | C2O2 | **133** | C6O3H13 |  | | **56** | C2O2 | |  | | **133** | C6O3H13 | |  | |
| 57 | C2O2H | **134** | C5O4H10 |  | | **57** | C2O2H | |  | | **134** | C5O4H10 | |  | |
| 58 | C2O2H2 | **135** | C5O4H11 |  | | **58** | C2O2H2 | |  | | **135** | C5O4H11 | |  | |
| 59 | C2O2H3 | **136** | C6O4 |  | | **59** | C2O2H3 | |  | | **136** | C6O4 | |  | |
| 60 | C2O2H4 | 137 | C6O4H |  | | **60** | C2O2H4 | |  | | **137** | C6O4H | |  | |
| 61 | C2O2H5 | **138** | C6O4H2 |  | | **61** | C2O2H5 | |  | | **138** | C6O4H2 | |  | |
| 62 | C2O2H6 | **139** | C6O4H3 |  | | **62** | C2O2H6 | |  | | **139** | C6O4H3 | |  | |
| 63 | C2O2H7 | **140** | C6O4H4 |  | | **63** | C2O2H7 | |  | | **140** | C6O4H4 | |  | |
| 64 | C4O | **141** | C6O4H5 |  | | **64** | C4O | |  | | **141** | C6O4H5 | |  | |
| 65 | C4OH | **142** | C6O4H6 |  | | **65** | C4OH | |  | | **142** | C6O4H6 | |  | |
| 66 | C4OH2 | **143** | C6O4H7 |  | | **66** | C4OH2 | |  | | **143** | C6O4H7 | |  | |
| 67 | C4OH3 | **144** | C6O5H8 |  | | **67** | C4OH3 | |  | | **144** | C6O5H8 | |  | |
| 68 | C3O2 | **145** | C6O4H9 |  | | **68** | C3O2 | |  | | **145** | C6O4H9 | |  | |
| 69 | C3O2H | **147** | C6O4H11 |  | | **69** | C3O2H | |  | | **147** | C6O4H11 | |  | |
| 70 | C3O2H2 | **148** | C6O4H12 |  | | **70** | C3O2H2 | |  | | **148** | C6O4H12 | |  | |
| 71 | C3O2H3 | **149** | C6O4H13 |  | | **71** | C3O2H3 | |  | | **149** | C6O4H13 | |  | |
| 72 | C3O2H4 | **150** | C6O4H14 |  | | **72** | C3O2H4 | |  | | **150** | C6O4H14 | |  | |
| 73 | (H2O)3(H30) | **151** | C5O5H11 |  | | **73** | (H2O)3(H30) | | | | **151** | C5O5H11 | |  | |
| 77 | C3O2H9 | **152** | C6O5 |  | | **77** | C3O2H9 | |  | | **152** | C6O5 | |  | |
| 78 | C5OH2 | **153** | C6O5H |  | | **78** | C5OH2 | |  | | **153** | C6O5H | |  | |
| 79 | C5OH3 | **157** | C6O5H5 |  | | **79** | C5OH3 | |  | | **157** | C6O5H5 | |  | |
| 80 | C4O2 | **163** | C6O5H11 |  | | **80** | C4O2 | |  | | **163** | C6O5H11 | |  | |
| 81 | C4O2H | **169** | C6O6H |  | | **81** | C4O2H | |  | | **169** | C6O6H | |  | |
| 82 | C4O2H2 | **171** | C6O6H3 |  | | **82** | C4O2H2 | |  | | **171** | C6O6H3 | |  | |
| 83 | C4O2H3 | **175** | C6O6H7 |  | | **83** | C4O2H3 | |  | | **175** | C6O6H7 | |  | |
| 84 | C4O2H4 | **181** | C6O6H13 |  | | **84** | C4O2H4 | |  | | **181** | C6O6H13 | |  | |
| 85 | C4O2H5 | **187** | C5O5H10+H2O+H3O | **85** | | | C4O2H5 |  | | **187** | | C5O5H10+H2O+H3O | | |  |
| 86 | C4O2H6 | **189** | C5O5H12+H2O+H3O | **86** | | | C4O2H6 |  | | **189** | | C5O5H12+H2O+H3O | | |  |
| 87 | C4O2H7 | **193** | C5O5H15+H3O+H3O | **87** | | | C4O2H7 |  | | **193** | | C5O5H15+H3O+H3O | | |  |
| 91 | (H2O)4(H30) | **199** | C6O6H12+H3O | **91** | | | (H2O)4(H30) | | | **199** | | C6O6H12+H3O | | |  |
| 92 | C6OH4 | **205** | C5O5H11+H2O+H2O+H2O | **92** | | | C6OH4 |  | | **205** | | C5O5H11+H2O+H2O+H2O | | |  |
| 93 | C6OH5 | **207** | C5O5H13+H2O+H2O+H2O | **93** | | | C6OH5 |  | | **207** | | C5O5H13+H2O+H2O+H2O | | |  |
| 94 | C6OH6 | **211** | C5O5H14+H3O+H3O+H3O | **94** | | | C6OH6 |  | | **211** | | C5O5H14+H3O+H3O+H3O | | |  |
| 95 | C6OH7 | **217** | C6O6H12+H2O+H3O | **95** | | | C6OH7 |  | | **217** | | C6O6H12+H2O+H3O | | |  |


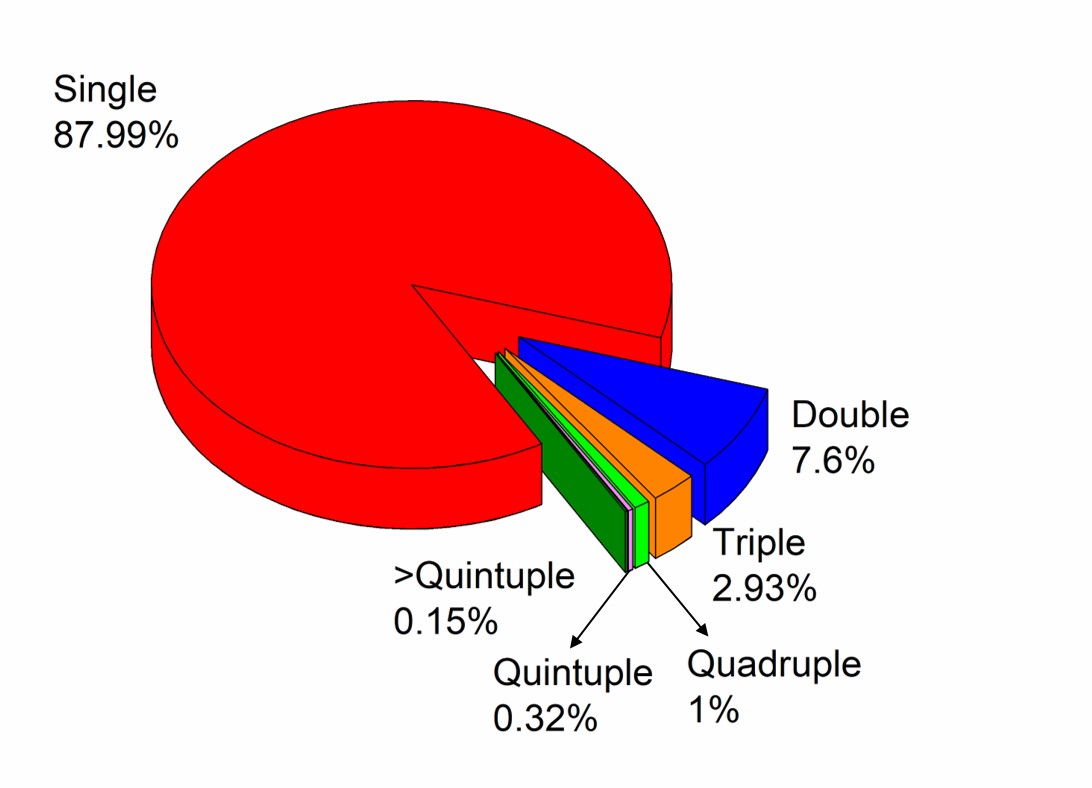


Figure A 1: The number of multi-hit events of the measurement in percentage.


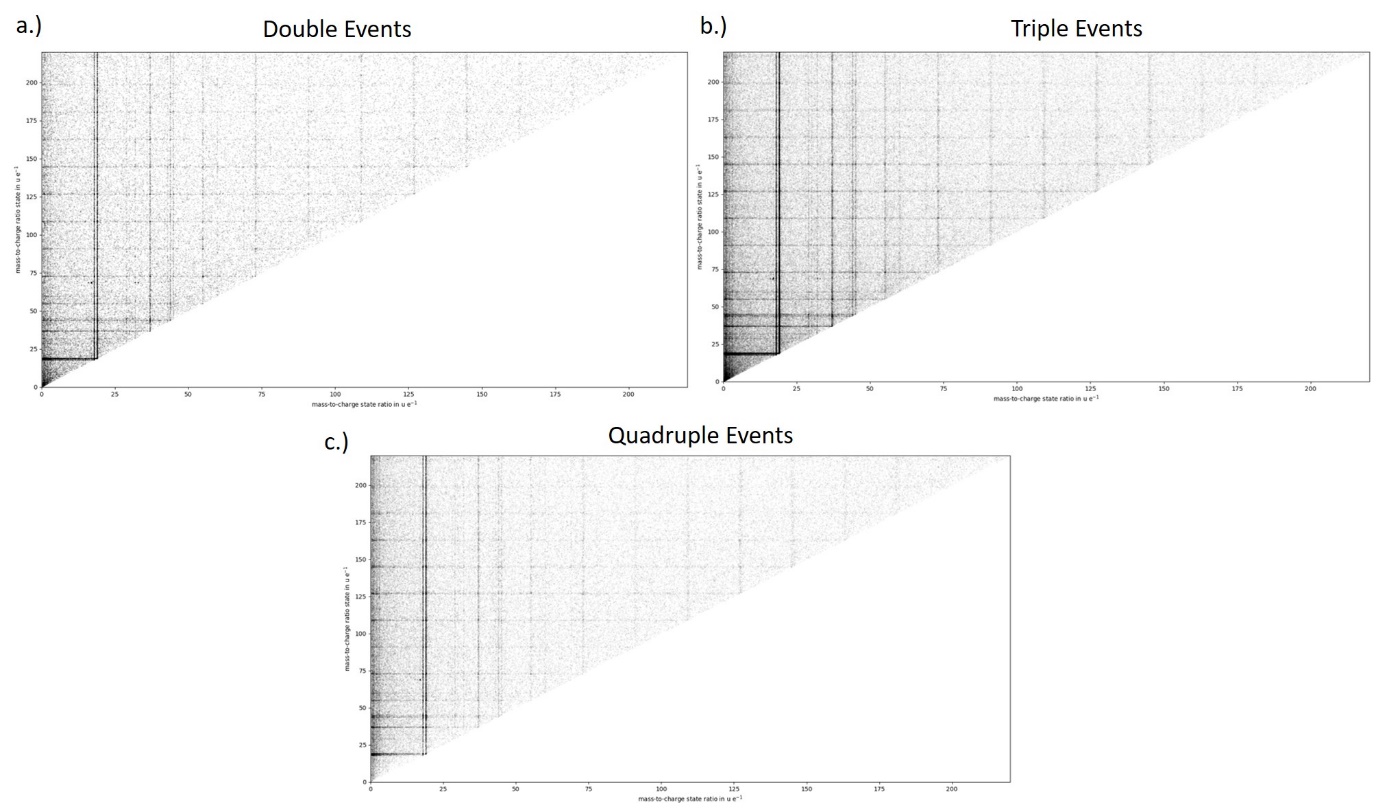


Figure A 2: a) Correlation histogramm for a supersaturated glucose solution, the measured mass-to-charge state ratio m_2_^’^/q_2_^’^ of the second event is plotted versus the measured mass-to-charge state ratio m_1_^’^/q_1_^’^ of the first event. In b.) the correlation histogram of triple events and in c.) for quadruple events are shown.


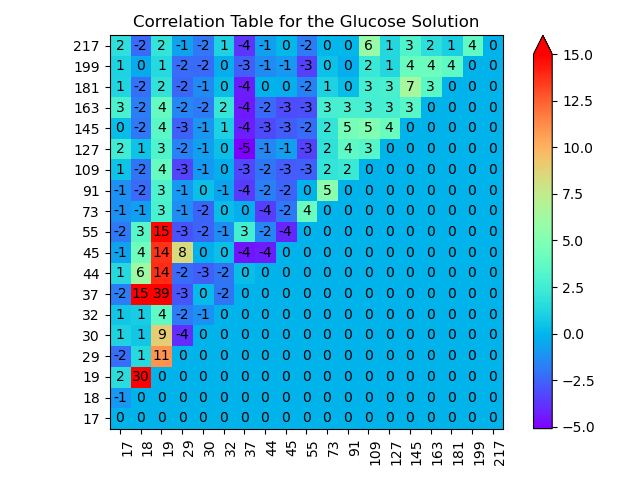


Figure A 3: Correlation table d_{ij}_ according to (32) for ion-pairs in the evaporation of glucose solution. The values represent the excess (or deficit) of the number of the co-evaporation events, compared to the uncorrelated co-evaporation of double events.


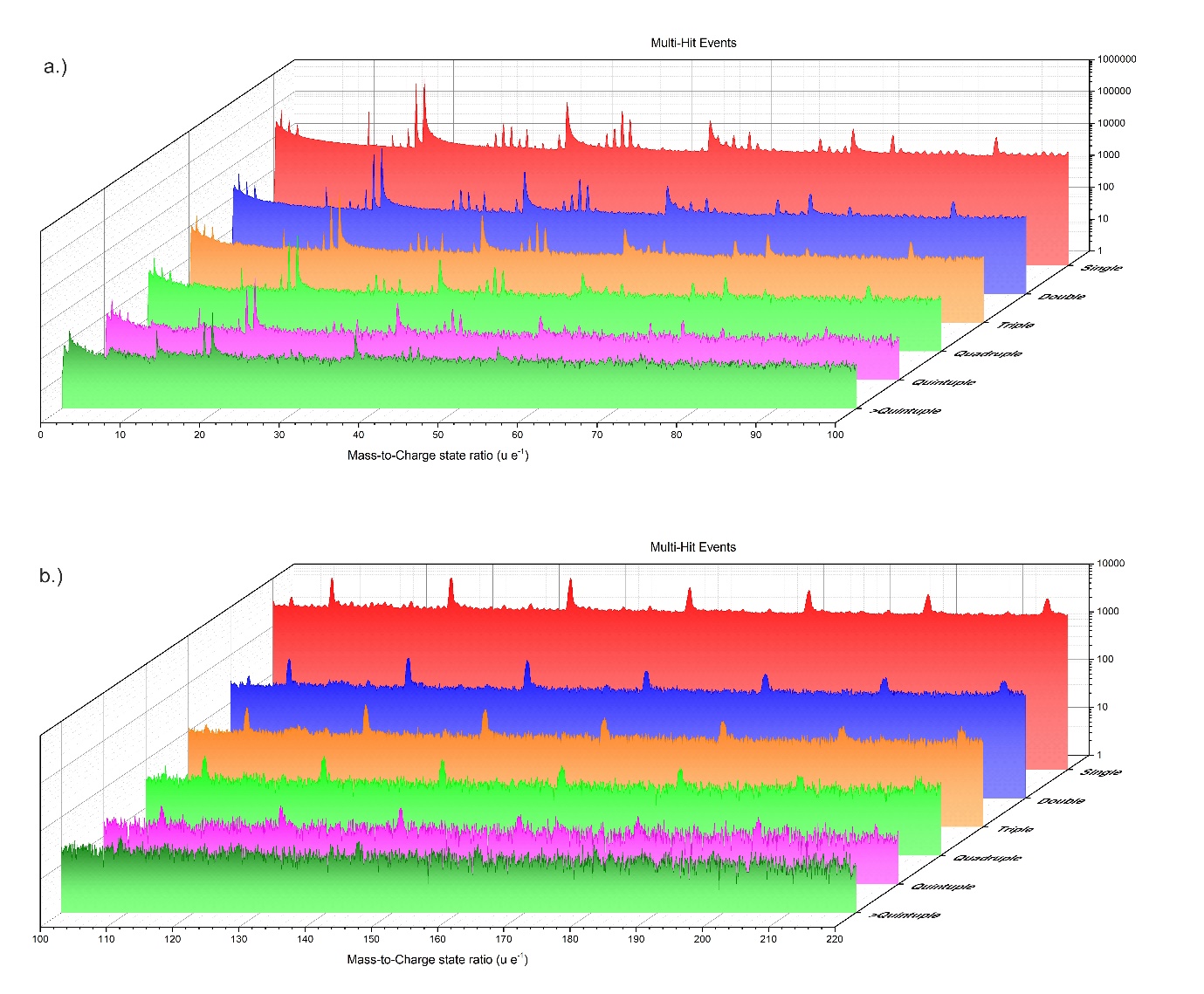


Figure A 4: Mass spectrum split into single, double, triple, quadruple, quintruple and >quintruple events in a.) from a mass-to-charge state ratio of 0-100 u e^-1^ and in b.) from 100-220 u e^-1^.


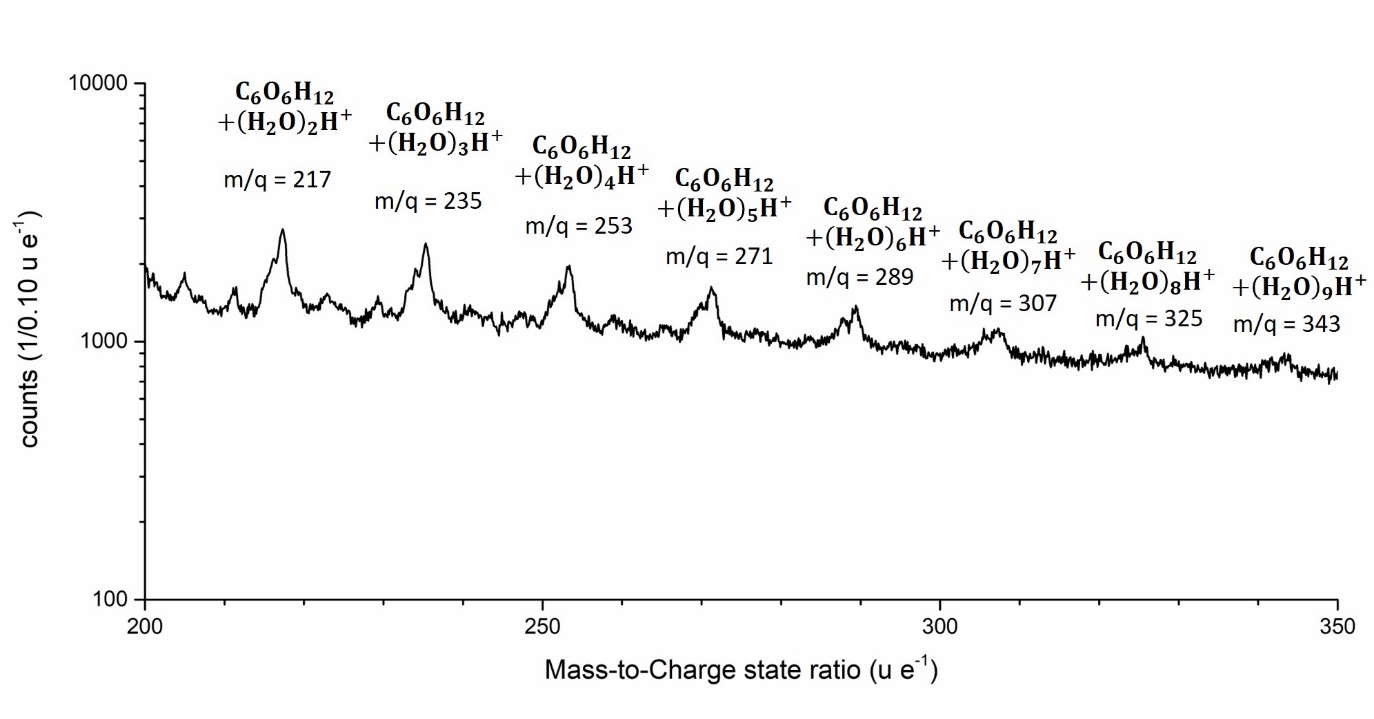


Figure A 5: Mass spectrum of a supersaturated glucose solution in a larger mass-to-charge state ratio from 200-350 u e^-1^ in a logarithmic plot. Larger glucose+water cluster with the formula C_6_O_6_H_12_+(H_2_O)_n_H^+^ with n = 2 – 9 are visible.
